# Supplementary material for: Do we need a core curriculum for medical students? A scoping review
Source: BMJ Open. 2019 Aug 30;9(8):e027369. doi: 10.1136/bmjopen-2018-027369 (PMC6720253; doi:10.1136/bmjopen-2018-027369)
Supplement: Supplementary data [file bmjopen-2018-027369supp001.pdf]

Example Search Strategy for Appendix

NICE Healthcare Databases Advanced Search MEDLINE 1946 to present (Provided by Proquest).

## **Strategy** 606888

| # | Database | Search term | Results |
|---|---|---|---|
| 1 | Medline | "EDUCATION, MEDICAL, UNDERGRADUATE"/ | 22398 |
| 2 | Medline | ("medical education").ti,ab | 36006 |
| 3 | Medline | (undergraduate).ti,ab | 29224 |
| 4 | Medline | ("medical student").ti,ab | 6528 |
| 5 | Medline | "STUDENTS, MEDICAL"/ | 30311 |
| 6 | Medline | (2 AND 3) | 3401 |
| 7 | Medline | (1 OR 4 OR 5 OR 6) | 48667 |
| 8 | Medline | CURRICULUM/ | 70249 |
| 9 | Medline | (curricul*).ti,ab | 48931 |
| 10 | Medline | (8 OR 9) | 93842 |
| 11 | Medline | (core OR influenc* OR design* OR develop* OR deliver* OR enabl* OR barrier* OR process* OR factor*).ti,ab | 9645339 |
| 12 | Medline | "REFERENCE STANDARDS"/ | 40365 |
| 13 | Medline | (standard*).ti,ab | 1110534 |
| 14 | Medline | (12 OR 13) | 1133896 |
| 15 | Medline | (11 OR 14) | 10194258 |
| 16 | Medline | DERMATOLOGY/ | 17224 |

| | | | |
|---|---|---|---|
| 17 | Medline | (dermatology).ti,ab | 23551 |
| 18 | Medline | (16 OR 17) | 33537 |
| 19 | Medline | exp "UNITED KINGDOM"/ | 350174 |
| 20 | Medline | (UK OR "United Kingdom" OR "Great Britain" OR GB OR England OR Wales OR "Northern Ireland" OR Scotland).ti,ab | 205051 |
| 21 | Medline | (19 OR 20) | 459845 |
| 22 | Medline | (7 AND 10 AND 15) | 8130 |
| 23 | Medline | 22 [DT 1994-2019] | 7323 |
| 24 | Medline | (7 AND 10 AND 15 AND 18) | 57 |
| 25 | Medline | 24 [DT 1994-2019] | 52 |
| 26 | Medline | (7 AND 10 AND 15 AND 21) | 788 |
| 27 | Medline | 26 [DT 1994-2019] | 718 |
| 28 | Medline | (7 AND 10 AND 15 AND 18 AND 21) | 8 |
| 29 | Medline | 28 [DT 1994-2019] | 8 |
