# Supplementary material for: Do we need a core curriculum for medical students? A scoping review
Source: BMJ Open. 2019 Aug 30;9(8):e027369. doi: 10.1136/bmjopen-2018-027369 (PMC6720253; doi:10.1136/bmjopen-2018-027369)
Supplement: Author's manuscript [file bmjopen-2018-027369.draft_revisions.pdf]

BMJ Open

BMJ Open is committed to open peer review. As part of this commitment we make the peer review history of every article we publish publicly available.

When an article is published we post the peer reviewers' comments and the authors' responses online. We also post the versions of the paper that were used during peer review. These are the versions that the peer review comments apply to.

The versions of the paper that follow are the versions that were submitted during the peer review process. They are not the versions of record or the final published versions. They should not be cited or distributed as the published version of this manuscript.

BMJ Open is an open access journal and the full, final, typeset and author-corrected version of record of the manuscript is available on our site with no access controls, subscription charges or pay-per-view fees (http://bmjopen.bmj.com).

If you have any questions on BMJ Open's open peer review process please email info.bmjopen@bmj.com

Do we need a core curriculum for medical students? A scoping review

Journal:	*BMJ Open*
Manuscript ID	bmjopen-2018-027369
Article Type:	Research
Date Submitted by the Author:	22-Oct-2018

Complete List of Authors:	Sharma, Maulina; University Hospitals of Derby and Burton NHS Foundation Trust, Dermatology; University of Nottingham School of Medicine, Murphy, Ruth; University of Nottingham School of Medicine Doody, Gillian; University of Nottingham School of Medicine, Medical Education

Keywords:	Undergraduate, MEDICAL EDUCATION & TRAINING, United Kingdom, Core, Curriculum

SCHOLARONE™
Manuscripts

Do we need a core-curriculum for medical students? A scoping review

Maulina Sharma, Ruth Murphy, Gillian A Doody

Medical Education Unit, University of Nottingham, School of Medicine, Nottingham, NG7 2UH, UK

Maulina Sharma, Consultant Dermatologist, Honorary (Consultant) Associate Professor University of Nottingham, School of Medicine, Department of Dermatology London Road Community Hospital Derby DE1 2QY, UK

Ruth Murphy, Consultant Dermatologist, University of Nottingham, School of Medicine, Nottingham, NG7 2UH, UK

Gillian A Doody, Dean of Medical Education, Medical Education Unit, University of Nottingham, School of Medicine, Nottingham, NG7 2UH, UK

Corresponding author:

M Sharma maulinas02@gmail.com

Tel no: 01332 254653

Word count: 2585

Keywords: core curriculum, medical undergraduates, UK

For peer review only

Summary

Do we need a core curriculum for medical students? A scoping review

Objective: The General Medical Council (GMC) recommends medical schools to develop and implement curricula that enable students to achieve learning outcomes required as graduates.UK medical schools follow the GMC's Outcomes for Graduates which can be viewed as generic. There are also plans for the GMC to introduce a national Medical Licencing Assessment (MLA) for medical graduates who want to practice medicine in the UK in 2022. With no standardised or unified undergraduate (UG) curriculum in UK, various specialities have expressed concerns about not being represented in medical schools and have developed specialty-specific core-curricula.

The aim of the scoping review was to identify learned bodies who have developed a core-curriculum for UK medical schools and highlight the drivers, gaps and future approaches to curricular development and implementation.

Methods: A literature search was conducted using online databases (EMBASE, MEDLINE, ERIC, HMIC, PubMed and CDSR) and online search engines (Google & Google Scholar, Department of Health, GMC and BMA) for relevant articles from 1996 to May 2017 (i.e. past 20 years). A methodological framework to map the key concepts of undergraduate medical curriculum was followed. Any national body relevant to UK medical undergraduates with a core-curriculum was included.

Results: A total of 1267 articles were identified, 30 articles were eligible for inclusion, detailing undergraduate curricula for 27 specialties: 17 clinical specialties, 6 foundation subjects and 4 based on professionalism.

European, national and regional expert bodies had developed these curricula and provided guidance to medical schools on minimum standards in terms of the knowledge and skills required.

Conclusions: This study provides the first comprehensive overview of the available national UG core-curricula. The review explores the need for UK medical schools to align and revise their curricula and standards..

For peer review only

For peer review only - http://bmjopen.bmj.com/site/about/guidelines.xhtml

Do we need a core curriculum for medical students? A scoping review

Introduction

The General Medical Council (GMC) in 2016, set out the Promoting Excellence document which outlines a set of standards for all stages of medical education and training, including undergraduate (UG) medical education.[1] It recommends that medical schools develop and implement curricula that enable students to achieve learning outcomes required as graduates. The GMC also plans to introduce a Medical Licencing Assessment (MLA) in 2022 applicable to medical graduates who want to practice medicine in the UK.[2] Medical schools in the UK currently organise their own curricula and assessments based on GMC's Outcomes for Graduates which can be viewed as generic.[3] Once the students successfully graduate, the GMC grants them licence to practice.

In the UK there is a growing need for development of medical generalists with a holistic approach to patients presenting with complex needs. A 'Future Hospital Commission' report (FHC), through the Royal College of Physicians (RCP) in 2013 promoted the re-introduction of generalism into medical training.[4] This means that medical education and training of doctors in UK needs to address the current and future demographic of patients

With the generalist trend, as well as plans to introduce the MLA for all medical graduates in the UK, medical schools may be influenced on what they teach and assess. There is currently no unified or standardized UG medical curriculum in the UK. Various specialities have expressed concerns about not being adequately in medical school curricula and have developed specialty-specific core-curricula.

Methods

We performed a scoping review using the methodological framework as outlined by Arksey and O'Malley.[5] The framework was used to; identify the specialties, or subjects, which recommended and had developed an undergraduate core-curriculum for UK medical schools; select and search for relevant studies; chart and collate the data and summarise the results.

For peer review only - http://bmjopen.bmj.com/site/about/guidelines.xhtml

A literature search was conducted using online databases (EMBASE, MEDLINE, ERIC, HMIC, PubMed and CDSR) and online search engines (Google & Google Scholar, Department of Health, GMC and BMA) for relevant articles from 1996 to May 2017 (i.e. the past 20 years). Online resources and search engines were then assessed between 01/03/2017 and to 19/05/2017

Keywords or terms used in searches were: medical student, medical education, undergraduate, core-curriculum, national (UK and individual countries e.g. England), standards and GMC.

Inclusion criteria: All published studies, reports or articles which had a recommended core-curriculum for a specialty or subject for UK medical undergraduates on a national level were eligible. Any national body, society, college or organisation specific and pertinent to UK medical undergraduate education was included. For curricula which had been revised or updated, the most recent (up to 19/05/17) were included.

Exclusion criteria: Articles pertaining to core-curricula for postgraduate courses and those not specific to UK medical undergraduates.

Patient and Public involvement: As this was a scoping review of available undergraduate core-curricula, patients or public were not involved.

Electronic search results were managed using a reference manager, with data collated and reported using PRISMA flow diagram (Figure 1) and an electronic data collection table (Table 1). Titles and abstracts of all citations were first screened by the author (MS), those that were not related to undergraduate curricula or the UK were excluded. Full articles were requested from the library, when it was not possible to eliminate them from reading either their title or abstract. The full text of all potentially eligible articles was then obtained and assessed against the inclusion criteria (MS and RM). Where the full text of an article was not available the authors were contacted to request a copy. Any ambiguities about whether or not a study met the inclusion criteria were resolved by a consensus between authors (MS, RM, and GD).

Results

The initial search identified a total of 1246 articles, after duplicates were removed (n=21). All articles were screened and 108 were initially highlighted as of interest. Of these, 78 were subsequently excluded, with reasons recorded. A total of 30 articles were then included in the qualitative synthesis, which comprised 27 specialties or subjects (Figure1). The name of

For peer review only - http://bmjopen.bmj.com/site/about/guidelines.xhtml

specialty, or subject, in alphabetical order, organisational body for developing the curricula, year of curriculum update, and whether studies were aligned to GMC learning outcomes were then extracted and recorded on an electronic data collection table (Table 1).

The key concepts derived were broadly grouped into the following: categories of core-curricula and their timing within the school course; organisations involved in development of the core-curricula; drivers for development of core-curricula; and curriculum overlaps and gaps.

Grouping of curricula and timing within the school course

The curricula developed and recommended for UK medical schools could be grouped into 3 main categories: clinical specialties (n=17) (e.g. surgery, dermatology); foundation subjects (n=6) (e.g. anatomy, pharmacology) and subjects related to professionalism (n=4) (e.g. communication skills) (Table 1).

Clinical specialty curricula were applicable during years of training e.g. paediatrics, obstetrics & gynaecology, psychiatry, surgery, acute medical care, resuscitation and end of life care.[6-12] Some specialties had developed a curricula with a system-based approach e.g. neurology and dermatology, whilst others targeted specific health issues, such as alcohol abuse, or inequality in health access.[13-16]

Foundation subjects like anatomy, pathology and nutrition as well as curricula related to professionalism, like medical ethics and law, were developed so as to be relevant throughout all medical school training.[17-20]

Organisations involved in development of individual core-curricula

Curricula were developed by international, national and regional organisations with participation from relevant stakeholders. Consensus agreement derived from a Delphi process was the preferred method of achieving core-curricular recommendations.[10,11,15,17,21-23]

Four curricula, namely communications skills, geriatrics, musculoskeletal system and palliative care had their core-curriculum co-developed by two different international and national bodies. The international recommendations encompassed the national guidance.[21-26]

For peer review only - http://bmjopen.bmj.com/site/about/guidelines.xhtml

National organisations, such as the Royal Colleges of the UK and national specialist consultant societies and bodies, developed 12 specialty specific curricula.[10,11,13,14,17,20,23,24,27,29-31]

National consultant expert consensus was used to provide guidelines for a core curriculum in urology, which was developed through a survey of consultant urologists, whilst a separate urology syllabus for medical undergraduates was developed by the British Association of Urological Surgeons.[30,31]

At a regional level, two medical schools developed and recommended curricula one for alcohol related disorders and the other for the medical humanities.[15,32]

Drivers for development of core-curricula

Patient safety

Patient safety was a common theme in the development of core-curricula for clinical specialties, foundation subjects, in addition to patient expectations of a graduate in terms of medical ethics and law. [20,22,23] The World Health Organisation (WHO), have published the multi-professional patient safety curriculum guide to aid teaching of patient safety in universities and schools, including medicine.[33] The ACUTE initiative identified the core set of competencies for resuscitation and care of acutely ill patients that all undergraduate trainees require to be competent in at the point of graduation.[10] This was designed to address the sub-optimal care of the critically ill in hospital where contributory factors included recognition of critical illness, lack of knowledge, a lack of supervision, failure to seek advice and poor communication.[10] Similarly, the British Pharmacology Society (BPS) prepared a curriculum to standardise the learning objectives and recommended introduction of prescribing safety assessments in all medical schools, in alignment with GMC's outcomes.[28,]

Disease burden and needs of society

In the UK, cancer affects 1 in 3 people.[34] The Royal College of Radiologists who developed the UG radiology curriculum also developed the non-surgical oncology curriculum. With collaboration with the Royal College of Physicians' Joint Collegiate Council for Oncology (JCCO), the UG curriculum with its joint approach aims to address competencies in a multidisciplinary approach towards patients affected with cancer and their needs.[34, 35]

For peer review only - http://bmjopen.bmj.com/site/about/guidelines.xhtml

Societal requirements of an ageing, frail population, with complex problems related to independence and mobility were important factors for the development of a core-curriculum for geriatric medicine and musculoskeletal system. [21, 24-6]

Alcohol is estimated to cost the UK public sector £20 billion a year and account for 7–40% of non A&E hospital admissions, 20% of primary healthcare consultations and 70% of A&E visits between midnight and 5am.[36] Undergraduate teaching about alcohol and its sequelae varies greatly, but may be no more only a few hours throughout a five year course. The new alcohol use disorders (AUD) curriculum reflects the need to produce a generation of doctors equipped to identify alcohol related problems and to reduce the projected consequences of alcohol abuse and the associated burden on the health service.[15]

Public healthcare prevention and promotion, human nutrition and its relevance to health and disease and healthcare inequalities all have specific UG curricula.[16,19, 27]

Lack of representation and inadequate preparedness amongst graduates

Multiple specialties, through national surveys across UK medical schools, felt a lack of adequate representation, teaching or assessments in medical school curricula.[14,29,37,38,39] These concerns were echoed by feelings of inadequacy amongst junior doctors and general practitioners due to a lack of training during undergraduate training in some specialties, some of these specialties have subsequently developed their own curricula.[29,38,40] This is particularly seen in specific core-curricula like ENT and Dermatology, where related diseases and conditions are common presentations in UK primary care.[14,37] This has led such specialties to develop specialty specific learning objectives, defining minimum knowledge and skills to be attained.[36,42]

Curriculum: overlaps and gaps

The General Medical Council, based on a review of curriculum theories, its relevance and context with regards to medical education and its stakeholders i.e. students, teachers and regulators, defines “a curriculum as a statement of the intended outcomes, encompassing content, teaching, learning and assessment methods, feedback and supervision as part of the educational programme”.[41]

For peer review only - http://bmjopen.bmj.com/site/about/guidelines.xhtml

This scoping review found that the Royal Colleges of UK, like the Royal College of Surgeons (RCS) and Obstetrics and Gynaecology (RCOG) covered the curricular requirements comprehensively by highlighting content and how to achieve the learning objectives with structure and assessment methods.[7,9]

However, many clinical and foundation subjects suggested medical schools determine how best to incorporate content within their medical course to allow flexibility in their implementation, delivery and assessment. Hence, they were focussed primarily on syllabus content. [14,17,30,37,42]

Despite representation of surgical sub-specialties like ENT and urology in the RCS curriculum, both sub-specialities developed specific outcomes for medical school curricula.[29,30,37] Overlapping of core-content was also seen between more established curricula like geriatrics[24] and psychiatry.[8] The Royal college of General Practitioners (RCGP), one of the largest royal colleges which sets out standards for family doctors in UK has recently produced a core-curriculum for UG students.[43] Interestingly, there is no specific UG curriculum for medicine even though the Royal College of Physicians (RCP), sets out standards for specialty physicians in UK.

Discussion

Though the GMC recommends a 'core curriculum' for UG medical education, medical educators have found it challenging to define what is meant by core, what needs to be included and its relevance to the bigger context of competency for graduating medical students.[1,2,3] Whilst medical schools align their curricula to the generic Outcomes for Graduates, explicit specialty representation and mapping of burden of disease in curricula has been challenging.[27,44,45] Furthermore, the introduction of a UK-wide Medical Licencing Assessment in 2022 may influence what medical schools teach and provide opportunities to keep curricular content relevant and inclusive. The good intentions of the developed core-curricula have been to provide guidance to medical schools and allow flexibility in implementation. The available specialty specific core-curricula have been developed through international and national expert consensus, advising on minimum standards on core knowledge and skills to be achieved by medical graduates and align themselves to the Outcomes for Graduates document. The 'core-knowledge' in specialty specific curricula could help develop minimum standards and provide

For peer review only - http://bmjopen.bmj.com/site/about/guidelines.xhtml

quality assurance for both generic and specialty-specific areas across all UK medical schools. As Voss et al have noted, 'Improving patient safety and quality in health care is one of medicine's most pressing challenges'.[46] The GMC has also highlighted these areas in the new Outcomes for Graduates.[3]

Medical schools have an enormous task of continuously responding to changes in clinical practice, keeping the curriculum in context and ensuring that students are not over burdened by content. In the review, it was observed that there was duplication and overlapping of some conditions and diseases between specialties and core-curricular recommendations. An integrated approach to combine specialties would help reduce content overload, whilst keeping teaching and learning contextual. The introduction of longitudinal integrated clerkship in medical schools is looking to address this issue.[47] In addition, further adoption of inter-professional education can help to change attitudes and behaviours and provide a sustainable model to accommodate large numbers of students.[48,49] For example, the non-surgical oncology and palliative care national curricula could be integrated and specialist nurses involved in helping medical students understand different aspects of cancer and end of life care.[11,12,35]

The curricula developed for foundation and professionalism related subjects recommended their curricular components be integrated both horizontally and vertically throughout the medical course.[17-22] These subjects, relevant and applicable to all clinical specialties, should be included in generic outcomes for the specialties and form a framework through the undergraduate years.

In 2016, the UK government announced strategies to fund, develop and grow the general practice workforce, to meet the increasing demands of an aging population with complex needs and chronic disease burden.[50] Medical schools need to prepare approximately half their graduates for careers in general practice.[43] The new UG curriculum for general practice has been developed to complement postgraduate curriculum reflecting how curricula have evolved in relation to health care service and societal needs.[43]

Thus, by examining the evidence for need to change or update medical curricula, and through reflection on content and process, Mezirow's concept of transformative learning could be applied by medical educators in curricular development.[51] For instance in Scotland, the Scottish Deans' Medical Curriculum Group (SDMCG) discusses and helps coordinate the development, delivery and evaluation undergraduate curricula. Their aim is to ensure that graduates from each school in Scotland are of an equivalent standard. Their review of the curriculum has also

For peer review only - http://bmjopen.bmj.com/site/about/guidelines.xhtml

been informed by frameworks on specialist subjects like acute and emergency medicine, dermatology, neurology, palliative care, pharmacology and sexual health.[52]

With limited availability of resources and time, the curricula could be split into essential, desirable and nice to know categories to help prioritise areas of teaching and learning. The use of technological advances (e.g. use and sharing of e-learning resources/ webinars) could help provide flexibility and autonomy in how these curricula could be implemented and delivered, if followed.

Limitations of the review

We conducted a comprehensive search for all relevant articles for this study. However, as with limitations seen in scoping reviews, articles or studies pertinent to the review question may have been unintentionally omitted.[3] The level of evidence for the quality of the developed specialty specific core-curricula could not be graded in this search, as done with systematic reviews. However, most curricula were developed with expert consensus and use of a Delphi study, suggesting that efforts were made to establish 'best practice' and core curricular outcomes. The curricular components for individual specialties were variable, making comparisons difficult.

We were nevertheless able to establish common themes and drivers for development of the UG core-curricula for UK medical undergraduates. In addition, the study helped identify gaps in specialty specific curricula like the absence of an overarching RCP curricula, as well as the heterogeneity of the curricular designs between specialties.

Conclusions

This literature review highlights the current status of specialty specific core curricula for medical graduates in UK. This is the first study to provide a comprehensive overview of the curricula developed by specialties and their recommendations on knowledge and skills expected of all UK medical graduates, in alignment with GMC. It would be interesting to see how medical schools are implementing and assessing these curricula and how that is likely to affect future training.

For peer review only - http://bmjopen.bmj.com/site/about/guidelines.xhtml

Contributorship statement:

All 3 authors have contributed to the planning, conduct, and reporting of the work described in the article, and MS is responsible for the overall content as guarantor. The corresponding author attests that all listed authors meet authorship criteria and that no others meeting the criteria have been omitted."

Funding: None

Conflicts of interest: None

Copyright/ Licence for publication:

The Corresponding Author has the right to grant on behalf of all authors and does grant on behalf of all authors, a worldwide licence to the Publishers and its licensees in perpetuity, in all forms, formats and media (whether known now or created in the future), to i) publish, reproduce, distribute, display and store the Contribution, ii) translate the Contribution into other languages, create adaptations, reprints, include within collections and create summaries, extracts and/or, abstracts of the Contribution, iii) create any other derivative work(s) based on the Contribution, iv) to exploit all subsidiary rights in the Contribution, v) the inclusion of electronic links from the Contribution to third party material where-ever it may be located; and, vi) licence any third party to do any or all of the above."

Acknowledgements:

For peer review only - http://bmjopen.bmj.com/site/about/guidelines.xhtml

Dr Rakesh Patel, Clinical Associate Professor in Medical Education & Honorary Consultant Nephrologist, Faculty of Medicine & Health Sciences, University of Nottingham for guidance on the review question

Lisa Lawrence, Clinical librarian, Burton and Derby Teaching Hospitals NHS Foundation Trust, Derby for conducting the searches for the review

Some of the work has been previously presented as oral presentation at:

British Association of Dermatologist (BAD) Teachers of Undergraduate Dermatology Annual Meeting on 6th March 2018

Association for the Study of Medical Education (ASME) Annual Scientific Meeting on 12th July 2018

Ethical approval: None required

For peer review only - http://bmjopen.bmj.com/site/about/guidelines.xhtml

For peer review only

For peer review only - http://bmjopen.bmj.com/site/about/guidelines.xhtml

References:

1. Promoting excellence: standards for medical education and training. (2015). Manchester, pp.6, 7. Available at: https://www.gmc-uk.org/-/media/documents/Promoting_excellence_standards_for_medical_education_and_training_0715.pdf_61939165.pdf [Accessed 28 Sep. 2018].

2. Medical Licencing Assessment. (2018). General Medical Council. Available at: https://www.gmc-uk.org/education/standards-guidance-and-curricula/projects/medical-licensing-assessment [Accessed 28 Sep. 2018].

3. Outcomes for graduates. (2018).General Medical Council. Available at: https://www.gmc-uk.org/-/media/documents/dc11326-outcomes-for-graduates-2018_pdf-75040796.pdf [Accessed 28 Sep. 2018].

4. Future Hospital: caring for medical patients. (2013). Available at: https://www.rcplondon.ac.uk/projects/outputs/future-hospital-commission [Accessed 28 Sep. 2018].

5. Arksey H, O'Malley L. Scoping studies: towards a methodological framework. Int J Soc Res Methodol. 2005; 8 (1):19-32.

For peer review only - http://bmjopen.bmj.com/site/about/guidelines.xhtml

6. The Royal College of Paediatrics. Undergraduate Curriculum for child health. 2015. http://www.rcpch.ac.uk.

[Accessed 3 Apr 2017].

7. Royal College of Obstetrics & Gynaecologists. National Undergraduate Curriculum in Obstetrics and Gynaecology.UK.2015. https://www.rcog.org.uk/en/careers-training/specialty-training-curriculum/undergraduate-training-in-og/

[Accessed 3 Apr 2017].

8. Royal College of Psychiatrists. Core Curriculum in Psychiatry. 2011. http://www.rcpsych.ac.uk/workinpsychiatry/faculties/academic/undergraduateteaching/curriculum.aspx.

[Accessed 4 Apr 2017].

9. The Royal College of Surgeons. National Undergraduate Curriculum in Surgery.2015.

https://www.rcseng.ac.uk//media/files/rcs/library-and-publications/non-journal-publications/undergraduate_curriculum_2015-web.pdf.

[Accessed 14 May 2017].

10. Baldock C. The Acute Care Undergraduate TEaching (ACUTE) Initiative: consensus development of core competencies in acute care for undergraduates in the United Kingdom. Intensive Care Med. 2005; 31 (12):1627-33.

For peer review only - http://bmjopen.bmj.com/site/about/guidelines.xhtml

11. Linklater GT, Bowden J, Pope L et al. Developing learning outcomes for medical students and foundation doctors in palliative care: a national consensus-seeking initiative in Scotland. Med Teach. 2014; 36 (5): 441-446.

12. Paes P, Wee B. A Delphi study to develop the Association for Palliative Medicine consensus syllabus for undergraduate palliative medicine in Great Britain and Ireland. Palliative medicine. 2008; 22 (4): 360-364.

13. Association of British Neurologists. Undergraduate Neurology.2017. http://www.theabn.org/what-we-do/training/undergraduate-neurology.html [Accessed 14 May 2017].

14. British Association of Dermatologists. Undergraduate Dermatology Curriculum.2016 http://www.bad.org.uk/librarymedia/documents/BAD%202016%20revised%20UG%20Curriculum%20(UK).pdf. Accessed 14 May 2017].

15. Steed H, Groome M, Rice P et al. Evaluation of a new core curriculum on alcohol use disorders for undergraduate medical students. Alcohol and alcoholism.2010; 45 (4):395-397.

16. Royal College of General Practitioners. A Core Curriculum for Learning About Health Inequalities in UK Undergraduate Medicine.2013. https://www.rcgp.org.uk. [Accessed 3 Apr 2017].

For peer review only - http://bmjopen.bmj.com/site/about/guidelines.xhtml

17. Smith CF, Finn GM, Stewart J, McHanwell S. Anatomical Society core regional anatomy syllabus for undergraduate medicine: The Delphi process. J.Anat. 2016; 228 (1):2-14.

18. The Royal College of Pathologists. Pathology Undergraduate Curriculum. 2014. https://www.rcpath.org/resourceLibrary/pathology-undergraduate-curriculum-.html.

[Accessed 3 Apr 2017].

19. Jackson, A.A., Human nutrition in medical practice: the training of doctors. Proc Nut Soc. 2001; 60 (2): 257-63.

20. Stirrat GM, Johnston C, Gillon R. Boyd K. Medical ethics and law for doctors of tomorrow: the 1998 Consensus Statement updated. J med ethics. 2010; 36 (1):55-60.

21. Masud T, Blundell A, Gordon AL, et al. European undergraduate curriculum in geriatric medicine developed using an international modified Delphi technique. Age and ageing. 2014; 43 (5):695-702.

22. Bachmann C, Abramovitch H, Barbu CG, et al. A European consensus on learning objectives for a core communication curriculum in health care professions. Patient Educ Couns. 2013; 93 (1):18-26.

23. von Fragstein M, Silverman J, Cushing A, et al. UK consensus statement on the content of communication curricula in undergraduate medical education. Med educ. 2008; 42 (11):1100-7.

24. British Geriatrics Society Recommended Curriculum in Geriatric Medicine for MedicalUndergraduates.2013.

For peer review only - http://bmjopen.bmj.com/site/about/guidelines.xhtml

http://www.bgs.org.uk/generalinformation171/secttrainees/generalinfo/undergraduatecurriculum.

[Accessed 14 May 2017].

25. Woolf AD, Walsh NE. Global core recommendations for a musculoskeletal undergraduate curriculum. Ann Rheum Dis. 2004; 63:517-24.

26. Coady D, Walker D, Kay L. Regional Examination of the Musculoskeletal System (REMS): a core set of clinical skills for medical students. Rheumatology. 2004; 43: 633–639.

27. Public Health Educators in Medical Schools. Undergraduate Public Health Curriculum for UK Medical Schools. Consensus Statement 2014.http://www.fph.org.uk/uploads/PHEMS booklet web.pdf. [Accessed 14 May 2017].

28. Ross S, Maxwell S. Prescribing and the core curriculum for tomorrow's doctors: BPS curriculum in clinical pharmacology and prescribing for medical students. Br. J. Clin. Pharmacol. 2012; 74 (4):644-61.

29. Turner K, Brewster S. Undergraduate urology: A survey of current provisions and guidelines for a core curriculum. BJU Int. 2002; 90 (4): 475.

30. The British Association of Urological Surgeons. An undergraduate syllabus for urology.2013. https://www.baus.org.uk/_userfiles/pages/files/professionals/education/Undergraduate%20Syllabus.pdf.

For peer review only - http://bmjopen.bmj.com/site/about/guidelines.xhtml

[Accessed 14 May 2017].

31. Core curriculum for sociology in undergraduate medical education. Behavioural & Social Sciences Teaching in Medicine (BeSST) Sociology SteeringGroup.2016.http://www.ed.ac.uk/usher/news-events/news-2016/core-curriculum-sociology-medical-education. [Accessed 20 Apr 2017].

32. Bleakley A, Marshall R, Brömer R. Toward an aesthetic medicine: developing a core medical humanities undergraduate curriculum. J med humanit. 2006; 27 (4):197-213.

33. WHO Multi-professional Patient Safety Curriculum Guide.2011. World Health Organization.http://www.who.int/patientsafety/education/mp_curriculum_guide/en/ [Accessed 3 Apr 2017].

34. The Royal College of Radiologists. Medical undergraduate non-surgical oncologycurriculum.2014. https://www.rcr.ac.uk/clinical-oncology/undergradute-oncology. [Accessed 4 May 2017].

35. The Royal College of Radiologists. Undergraduate radiology curriculum second edition. London, 2017. www.rcr.ac.uk. [Accessed 4 Apr 2017].

36. Royal College of Physicians. (2001) Alcohol - Can the NHS Afford It? London: RCP. pp 1–58.

37. Lyoyd S, Tan ZE, Taube M-A, Doshi J. Development of an ENT undergraduate curriculum using a Delphi survey. Clin Otolaryngol. 2014; 39:281-288.

38. Burge SM. Undergraduate medical curricula: are students being trained to meet future service needs? Clin Med.2003; 3 (3): 243-6.

For peer review only - http://bmjopen.bmj.com/site/about/guidelines.xhtml

39. Association of British Neurologists Training & Education Subcommittee. Clinical Neurology Medical Undergraduate Curriculum UK Medical School Survey.2002. http://www.theabn.org/media/Med%20Sch%20Neuro%20Survey%202002-3.pdf [Accessed 30/7/14] and CM Wiles, P Bain (Personal communications 2011).

40. Wilkinson IMS. A survey of undergraduate teaching of clinical neurology in the UK 1990. J Neurol Neurosurg Psych. 1991; 54:266-68.

41. General Medical Council (2010) Standards for curricula and assessment systems. 2010. http://www.gmc-uk.org/education/postgraduate/standards_forcurricula_and _assessment_systems.asp. [Accessed 14 May 2017].

42. Royal College of Ophthalmologists. "Eyes & Vision Curriculum" for Undergraduate and Foundation Doctors. 2015. https://www.rcophth.ac.uk/wp-content/uploads/2014/07/Undergraduate-and-Foundation-doctors-curriculum.pdf.[Accessed 3 Apr 2017].

43. Royal College of General Practitioners. (2018). Undergraduate teaching. http://www.rcgp.org.uk/training-exams/discover-general-practice/teaching-general-practice.aspx[Accessed 10 Oct 2018].

44. Wylie A, Thompson S. Establishing health promotion in the modern medical curriculum: a case study. Med Teach.2007; 29 (8):766-71.

45. McIntosh N, Olver RE, Haddad, D. What is core? Guidelines for the core curriculum in paediatrics. Med Educ.1997; 31(5): 354-8.

For peer review only - http://bmjopen.bmj.com/site/about/guidelines.xhtml

46. Voss JD, May NB, Schorling JB et al. Changing conversations: teaching safety and quality in residency training. Academic Medicine.2008; 83:1080-7.

47. Hirsh D, Gaufberg E, Ogur B, Cohen P, Krupat E, Cox M, Pelletier S, Bor D. Educational outcomes of the Harvard Medical School-Cambridge integrated clerkship: a way forward for medical education. Acad Med. 2012. 87(5): 643-50.

48. Ponzer S, Hylin U, Kusuoffsky A et al. Interprofessional training in the context of clinical practice: goals and students' perceptions on clinical education wards. Medical Education. 2004; 38(7):727-36.

49. Anderson ES, Lennox A. The Leicester Model of Interprofessional education: developing, delivering and learning from student voices for 10 years. J Interprof Care.2009; 23(6):557-73.

50. NHS England. General Practice Forward View.2016. https://www.england.nhs.uk/gp/gpfv/ [Accessed 22 May 2017].

51. Mezirow J and associates (eds). Fostering Critical Reflection in Adulthood. 1990. Jossey-Bass, San Francisco, CA.

52. Scottish Deans' Medical Curriculum Group. The Scottish Doctor: Learning Outcomes for the medical undergraduate in Scotland: a Foundation for Competent and Reflective Practitioners. 2008: Dundee. UK. http://www.scottishdoctor.org/resources/scotdoc3.pdf

[Accessed 22 May 2017].

For peer review only

For peer review only - http://bmjopen.bmj.com/site/about/guidelines.xhtml

For peer review only

For peer review only - http://bmjopen.bmj.com/site/about/guidelines.xhtml

Figure 1: PRISMA (preferred reporting items for systematic reviews and meta-analysis) flow diagram of the scoping review

UG= undergraduate

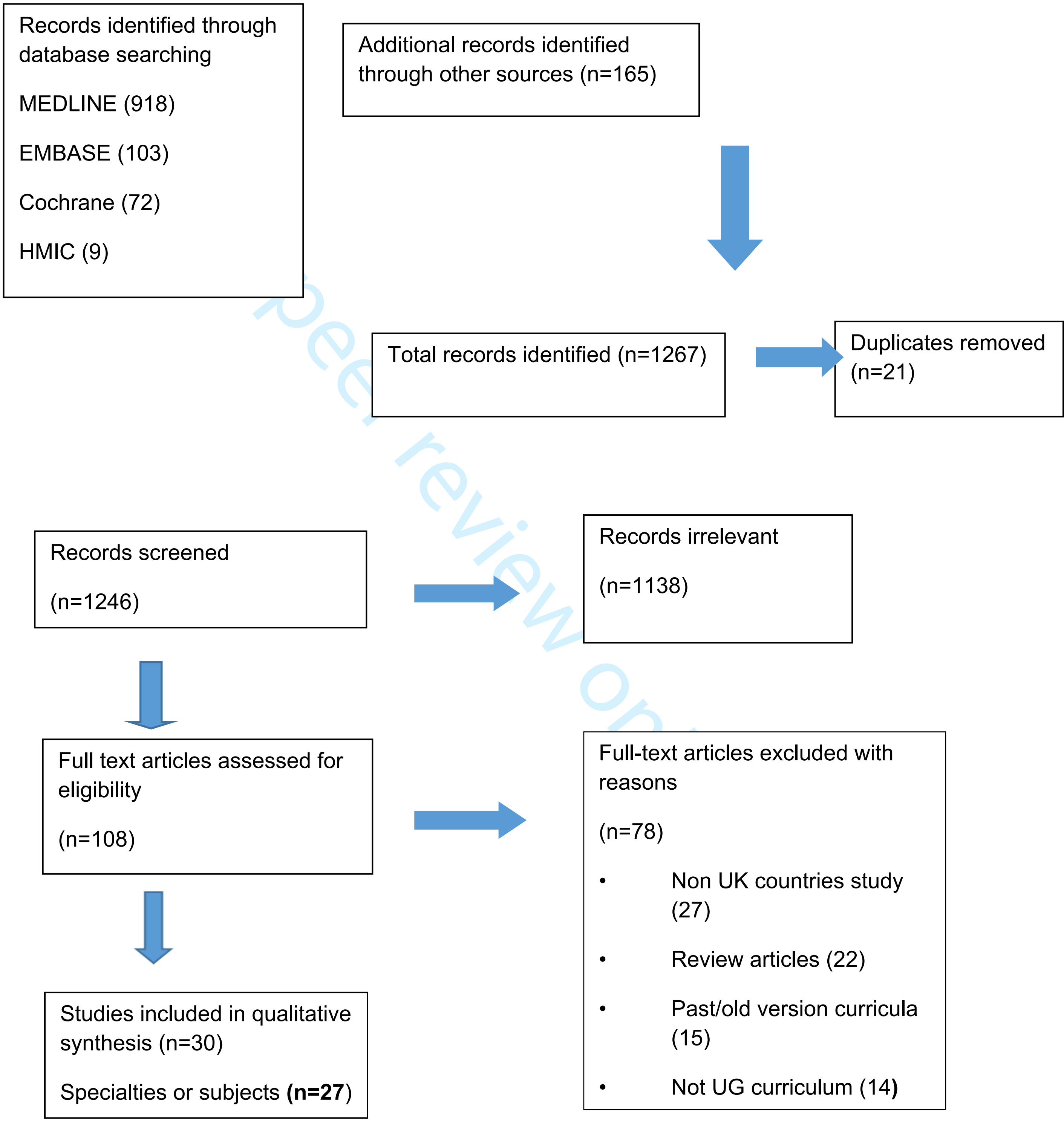

For peer review only - http://bmjopen.bmj.com/site/about/guidelines.xhtml

For peer review only

For peer review only - http://bmjopen.bmj.com/site/about/guidelines.xhtml

Table 1

	Specialty with curriculum	Year	Organization/Body	Alignment with GMC outcomes
**1.**	**Acute care**[10]	2005	Resuscitation Council UK	Y
**2.**	**Alcohol Use Disorders**[15]	2010	Ninewells Hospital and medical school, Dundee	Unclear
**3.**	Regional **Anatomy**[17]	2016	Anatomical Society	Y
**4.**	**Clinical pharmacology and prescribing**[28]	2012	British Pharmaceutical Society	Y
**5. a**	**Core communications curriculum**[22]	2013	European study using Delphi process	
**b**	**Communications curricula**[23]	2008	U. K. Council for Clinical Communication Skills Teaching in Undergraduate Medical Education	Y
**6.**	**Dermatology**[14]	2016	British association of dermatologists	Y
**7.**	**ENT**[37]	2014	A Delphi survey (UK) of ENT consultants and specialist registrars, accident and emergency consultants and specialist registrars, general practitioners and paediatricians.	Y
**8. a**	European undergraduate curriculum in **geriatric medicine**[21]	2014<br><br>2013	European Union of Medical Specialists	Y

For peer review only - http://bmjopen.bmj.com/site/about/guidelines.xhtml

b	**Geriatric medicine** for undergraduates[24]		British Geriatrics Society	Y
**9.**	**Health Inequalities in UK**[16]	2013	Royal college of general practitioners	Y
**10.**	**Medical Ethics and Law**[20]	2010	Medical Education Working Group of the Institute of Medical Ethics and associated signatories	Y
**11.**	**Medical Humanities**[32]	2006	Peninsula medical school	Unclear
**12. a**<br><br>b	**Musculoskeletal system (MSK)**[25]<br><br>**Regional Examination of the Musculoskeletal System (REMS)**[26]	2004<br><br>2004	The Bone and Joint Decade (BJD) Undergraduate Curriculum Development Group<br><br>UK representation of the four specialties- Rheumatologists, Orthopaedic Surgeons, Geriatricians and General Practitioners	Y
**13.**	**Neurology**[13]	2017	Association of British Neurologists	Y
**14.**	Human **Nutrition**[19]	2001	National Nutrition Task Force	Y
**15.**	**Obstetrics & Gynaecology**[7]	2015	Royal College of Obstetricians and Gynaecologists (RCOG)	Y
**16.**	**Ophthalmology**[42]	2015	Royal college of Ophthalmologists	Y
**17.**	**Oncology**[34]	2014	The Royal College of Radiologists	Y
**18.**	**Paediatrics**[6]	2015	Royal college of paediatrics and child	Y

For peer review only - http://bmjopen.bmj.com/site/about/guidelines.xhtml

			health	
**19. a**	**Palliative Care**[11]	2014	Scottish Palliative Medicine Curriculum Development Group	Y
**b**	**Palliative Medicine**[12]	2008	Association for Palliative Medicine	Y
**20.**	**Pathology**[18]	2014	The Royal College of Pathologists	Y
**21.**	**Patient Safety**[33]	2011	World Health Organization	Y
**22.**	**Psychiatry**[8]	2011	Royal college of psychiatrists	Y
**23.**	**Public Health**[27]	2014	Faculty of Public Health	Y
**24.**	**Radiology**[35]	2017	Royal college of radiologists	Y
**25.**	**Sociology**[31]	2016	Behavioural & Social Sciences Teaching in Medicine (BeSST) Sociology Steering Group	Y
**26.**	**Surgery**[9]	2015	Royal college of Surgeons	Y
**27. a**	**Urology**[29]	2002	Journal article in Urology	Unclear
**b**	**Urology syllabus**[30]	2012	British Association of Urological Surgeons	

For peer review only - http://bmjopen.bmj.com/site/about/guidelines.xhtml

Strengths and limitations of the study

- The first scoping review of the literature to identify the national specialty-specific and generic core-curricula developed and recommended for medical undergraduates in the UK
- The review establishes common themes and drivers for development of core-curricula for UK medical undergraduates
- The study identifies gaps in development of specific UG curricula like general medicine, as well as the heterogeneity of the curricular designs between specialties and subjects
- As with limitations seen in scoping reviews, articles or studies pertinent to the review question may have been unintentionally omitted

For peer review only

BMJ Open

DO WE NEED A CORE-CURRICULUM FOR MEDICAL STUDENTS? A SCOPING REVIEW

Journal:	*BMJ Open*
Manuscript ID	bmjopen-2018-027369.R1
Article Type:	Research
Date Submitted by the Author:	25-Mar-2019
Complete List of Authors:	Sharma, Maulina; University of Nottingham Faculty of Medicine and Health Sciences, Medical Education; University Hospitals of Derby & Burton NHS Foundation Trust , Dermatology<br>Murphy, Ruth; University of Nottingham School of Medicine<br>Doody, Gillian; University of Nottingham School of Medicine, Medical Education
**Primary Subject Heading**:	Medical education and training
Secondary Subject Heading:	Medical education and training
Keywords:	Undergraduate, MEDICAL EDUCATION & TRAINING, United Kingdom, Core, Curriculum

SCHOLARONE™
Manuscripts

DO WE NEED A CORE-CURRICULUM FOR MEDICAL STUDENTS? A SCOPING REVIEW

Maulina Sharma, Ruth Murphy, Gillian A Doody

Medical Education Unit, University of Nottingham, School of Medicine, Nottingham, NG7 2UH, UK

Maulina Sharma, Consultant Dermatologist, Honorary (Consultant) Associate Professor University of Nottingham, School of Medicine, Department of Dermatology London Road Community Hospital, Derby DE1 2QY, UK

Ruth Murphy, Consultant Dermatologist, University of Nottingham, School of Medicine, Nottingham, NG7 2UH, UK

Gillian A Doody, Dean of Medical Education, Medical Education Unit, University of Nottingham, School of Medicine, Nottingham, NG7 2UH, UK

Corresponding author:

M Sharma msxms16@nottingham.ac.uk

Tel no: 01332 254653

Word count: 2976

Keywords: core curriculum, medical undergraduates, UK

For peer review only - http://bmjopen.bmj.com/site/about/guidelines.xhtml

ABSTRACT

Objective

The General Medical Council (GMC) recommends medical schools to develop and implement curricula that enable students to achieve learning outcomes required as graduates.UK medical schools follow the GMC's Outcomes for Graduates which can be viewed as generic. There are also plans for the GMC to introduce a national Medical Licencing Assessment (MLA) for medical graduates who want to practice medicine in the UK in 2022. With no standardised or unified undergraduate (UG) curriculum in UK, various specialities have expressed concerns about not being represented in medical schools and have developed specialty-specific core-curricula.

The aim of the scoping review was to identify learned bodies who have developed a core-curriculum for UK medical schools and highlight the drivers, gaps and future approaches to curricular development and implementation.

Methods

A literature search was conducted using online databases (EMBASE, MEDLINE, ERIC, HMIC, PubMed and CDSR) and online search engines (Google & Google Scholar, Department of Health, GMC and BMA) for relevant articles from 1996 to May 2017 (i.e. past 20 years) and the search updated for any relevant articles up to 5th March 2019. A methodological framework to map the key concepts of undergraduate medical curriculum was followed. Any national body relevant to UK medical undergraduates with a core-curriculum was included.

Results

A total of 1283 articles were identified, 31 articles were eligible for inclusion, detailing undergraduate curricula for 26 specialties: 18 clinical specialties, 4 foundation subjects, 4 based on professionalism

European, national and regional expert bodies had developed these curricula and provided guidance to medical schools on minimum standards in terms of the knowledge and skills required.

Conclusions

For peer review only - http://bmjopen.bmj.com/site/about/guidelines.xhtml

This study provides the first comprehensive overview of the available national UG core-curricula. The review explores the need for UK medical schools to align and revise their curricula and standards.

Strengths and limitations of the study

- The first scoping review of the literature to identify the national specialty-specific and generic core-curricula developed and recommended for medical undergraduates in the UK.
- The review establishes common themes and drivers for development of core-curricula for UK medical undergraduates.
- The study identifies gaps in development of specific UG curricula like general medicine, as well as the heterogeneity of the curricular designs between specialties and subjects.
- As with limitations seen in other scoping reviews, articles or studies pertinent to the review question may have been unintentionally omitted.

For peer review only

For peer review only - http://bmjopen.bmj.com/site/about/guidelines.xhtml

INTRODUCTION

The General Medical Council (GMC) in 2016, set out the Promoting Excellence document which outlines a set of standards for all stages of medical education and training, including undergraduate (UG) medical education.[1] These standards should ensure that that medical schools develop and implement curricula to enable students to achieve learning outcomes required as graduates and practise safely and competently. The GMC also plans to introduce a Medical Licencing Assessment (MLA) in 2022 applicable to medical graduates who want to practice medicine in the UK.[2] Specialities have expressed concern about medical curricula as medical schools in the UK currently organise their own curricula and assessments based on GMC's Outcomes for Graduates which can be viewed as generic.[3] This may result in scanty representation of specialist knowledge and skills within the curriculum. Once the students successfully graduate, the GMC grants them licence to practice.

In the UK there is a growing need for development of medical generalists, that is, those who treat all common medical conditions and refer patients into secondary care for urgent or specialist treatment. A 'Future Hospital Commission' report (FHC), through the Royal College of Physicians (RCP) in 2013 promoted the re-introduction of generalism into medical training.[4] This means that medical education and training of doctors in UK needs to address the current and future demographic of patients.

With the generalist trend, as well as plans to introduce the MLA for all medical graduates in the UK, medical schools may be influenced on what they teach and assess. There is currently no unified or standardized UG medical curriculum in the UK. Various specialities have expressed concerns about not being adequately in medical school curricula and have developed specialty-specific core-curricula.

Our objective was to perform a scoping review of literature produced by learned bodies who have developed a core-curriculum for UK medical schools and to use this information to highlight the drivers, gaps and future approaches to curricular development and implementation.

For peer review only - http://bmjopen.bmj.com/site/about/guidelines.xhtml

METHODS

We performed a scoping review using the methodological framework as outlined by Arksey and O'Malley.[5] The framework was used to; identify the specialties, or subjects, which recommended and had developed an undergraduate core-curriculum for UK medical schools; select and search for relevant studies; chart and collate the data and summarise the results.

Search strategy

A literature search was conducted using online databases (EMBASE, MEDLINE, HMIC (searched via NICE Healthcare Databases Advanced Search), PubMed, The Cochrane Library and ERIC (Education Resources Information Centre). Other online search engines (Google & Google Scholar) were then searched as well as the Department of Health, GMC and BMA websites for relevant articles from 1996 to May 2017 (i.e. the past 20 years). The literature search was updated till 5th March 2019 for any further recent relevant articles.

Keywords or terms used in the searched included: General medical council (GMC), medical student, medical education, undergraduate, curriculum, standards and national (UK and individual countries e.g. England). An example of search strategy is included in Appendix 1.

Electronic search results were managed using a reference manager. Titles and abstracts of all citations were first screened by the author (MS), those that were not related to undergraduate curricula or the UK were excluded. Full articles were requested from the library, when it was not possible to eliminate them from reading either their title or abstract. The full text of all potentially eligible articles was then obtained and assessed against the inclusion criteria (MS and RM). Where the full text of an article was not available the authors were contacted to request a copy. Any ambiguities about whether or not a study met the inclusion criteria were resolved by a consensus between authors (MS, RM, and GD).

Eligibility criteria

For peer review only - http://bmjopen.bmj.com/site/about/guidelines.xhtml

Inclusion criteria were all published studies, reports or articles which had a recommended core-curriculum for a specialty or subject for UK medical undergraduates on a national level were eligible. Any national body, society, college or organisation specific and pertinent to UK medical undergraduate education was included. For curricula which had been revised or updated, the most recent (up to 05/03/19) were included.

Exclusion criteria were articles pertaining to core-curricula for postgraduate courses and those not specific to UK medical undergraduates. Due to the study being relevant to UK setting, non-English language literature was excluded.

Patient and Public involvement: As this was a scoping review of available undergraduate core-curricula, patients or public were not involved.

Data management

Data were collated and reported using PRISMA flow diagram (Figure 1). Characteristics of each study were recorded in an electronic data collection table and included the name of specialty, or subject, in alphabetical order, organisational body for developing the curricula, year of curriculum update, and whether studies were aligned to GMC learning outcomes were then extracted and recorded on an electronic data collection table (Table 1).

The key concepts derived were broadly grouped into the following: categories of core-curricula and their timing within the school course; organisations involved in development of the core-curricula; drivers for development of core-curricula; and curriculum overlaps and gaps.

RESULTS

The initial search identified a total of 1283 articles, after duplicates were removed (n=21). All articles were screened and 126 were initially highlighted as of interest. Of these, 95 were subsequently excluded, with reasons recorded. A total of 31 articles, published since 2002, and describing core curricula recommendations for medical undergraduates were then included in the narrative synthesis, which comprised 26 specialties or subjects (Figure1).

For peer review only - http://bmjopen.bmj.com/site/about/guidelines.xhtml

Narrative synthesis of results

Having identified publications from learned bodies who have developed a core-curriculum for UK medical schools, we identified the following themes and drivers for curricular development and implementation and summarised areas where there are gaps and overlaps which may influence future approaches to curricular development and implementation.

i) Grouping of curricula and timing within the school course

The curricula developed and recommended for UK medical schools could be grouped into 3 main categories: clinical specialty based (n=18) (e.g. surgery, dermatology); foundation subjects (n=4) (e.g. anatomy, pharmacology) and subjects related to professionalism (n=4) (e.g. communication skills) (Table 1).

Clinical specialty curricula were applicable during clinical years of training e.g. paediatrics, obstetrics & gynaecology, psychiatry, surgery, acute medical care, resuscitation and end of life care.[6-12] Some specialties had developed a curricula with a system-based approach e.g. neurology and dermatology.[13,14] Foundation subjects like anatomy, pathology and nutrition as well as curricula related to professionalism, like medical ethics and law, were developed so as to be relevant throughout all medical school training.[15-18]

ii) Organisations involved in development of individual core-curricula

Curricula were developed by international, national and regional organisations with participation from relevant stakeholders. Consensus agreement derived from a Delphi process was the preferred method of achieving core-curricular recommendations.[10, 11, 15, 18,-21,]

Four curricula, namely communications skills, geriatrics, musculoskeletal system and palliative care had their core-curriculum co-developed by two different international and national bodies. The international recommendations encompassed the national guidance.[19-24]

For peer review only - http://bmjopen.bmj.com/site/about/guidelines.xhtml

The Royal Colleges of UK developed specialty specific core-curricula for 9 specialties (e.g. general practice, pathology, radiology, surgery) [6-9, 16, 25-28]

National specialist consultant societies and bodies, developed 14 specialty specific curricula (e.g. dermatology, medical ethics, palliative care) [10—15, 17, 18, 21-24, 29, 30, 31].

National consultant expert consensus was used to provide guidelines for a core curriculum in urology and ENT, which was developed through a survey of consultants whilst a separate urology syllabus for medical undergraduates was developed by the British Association of Urological Surgeons.[32-34] At a regional level, one medical school developed and recommended a curriculum for medical humanities.[35]

i) Drivers for development of core-curricula

a) Patient safety

Patient safety was a common theme in the development of core-curricula for clinical specialties, foundation subjects, in addition to patient expectations of a graduate in terms of medical ethics and law. [15,18,21,27,22,23] The World Health Organisation (WHO), have published the multi-professional patient safety curriculum guide to aid teaching of patient safety in universities and schools, including medicine.[36] The ACUTE initiative identified the core set of competencies for resuscitation and care of acutely ill patients that all undergraduate trainees require to be competent in at the point of graduation.[10] This was designed to address the sub-optimal care of the critically ill in hospital where contributory factors included recognition of critical illness, lack of knowledge, a lack of supervision, failure to seek advice and poor communication.[10] Similarly, the British Pharmacology Society (BPS) prepared a curriculum to standardise the learning objectives and recommended introduction of prescribing safety assessments in all medical schools, in alignment with GMC's outcomes.[30,]

b) Disease burden and needs of society

For peer review only - http://bmjopen.bmj.com/site/about/guidelines.xhtml

In the UK, cancer affects 1 in 3 people.[26] The Royal College of Radiologists who developed the UG radiology curriculum also developed the non-surgical oncology curriculum. With collaboration from the Royal College of Physicians' Joint Collegiate Council for Oncology (JCCO), the UG curriculum with its joint approach aims to address competencies in a multidisciplinary setting towards patients affected with cancer and their needs.[26, 27]

Societal requirements of an ageing, frail population, with complex problems related to independence and mobility were important factors for the development of a core-curriculum for geriatric medicine and musculoskeletal system. [22-24]

The National Health Service of UK is underpinned by General Practitioners (GP) in UK, who remain the first point of contact for patients and provide long-term continuity of care. To provide a sustainable service to patients, UK needs approximately half of medical school graduates to pursue a career in family medicine or general practice.[25] In 2018, the Royal College of General Practitioners (RCGP) with Society for Academic Primary Care (SAPC) collaborated to develop the GP curriculum as a guidance to UK medical schools on the design and delivery of teaching of general practice to help bridge the gap between societal demands and needs. [25]

Public healthcare prevention and promotion, human nutrition and its relevance to health and disease also had specific UG curricula.[17, 25]

c) Lack of representation and inadequate preparedness amongst graduates

Multiple specialties, through national surveys across UK medical schools, felt a lack of adequate representation, teaching or assessments in medical school curricula.[13,14,32-34,37] These concerns were echoed by feelings of inadequacy amongst junior doctors and general practitioners due to a lack of training during undergraduate training in some specialties, some of these specialties have subsequently developed their own curricula.[13,38,39] This is particularly seen in specific core-curricula like ENT and Dermatology, where related diseases and conditions are common presentations in UK primary care.[14,34] This has led such specialties to

For peer review only - http://bmjopen.bmj.com/site/about/guidelines.xhtml

develop specialty specific learning objectives, defining minimum knowledge and skills to be attained.

iv) Curriculum: overlaps and gaps

The General Medical Council, based on a review of curriculum theories, its relevance and context with regards to medical education and its stakeholders i.e. students, teachers and regulators, defines “a curriculum as a statement of the intended outcomes, encompassing content, teaching, learning and assessment methods, feedback and supervision as part of the educational programme”.[40]

This scoping review found that the Royal Colleges of UK, like the Royal College of Surgeons (RCS) and Obstetrics and Gynaecology (RCOG) covered the curricular requirements comprehensively by highlighting content and how to achieve the learning objectives with structure and assessment methods.[7, 9]

However, many clinical and foundation subjects suggested medical schools determine how best to incorporate content within their medical course to allow flexibility in their implementation, delivery and assessment. Hence, they were focussed primarily on syllabus content. [13,14,28,34]

Despite representation of surgical sub-specialties like ENT and urology in the RCS curriculum, both sub-specialities developed specific outcomes for medical school curricula.[32-4] Overlapping of core-content was also seen between more established curricula like geriatrics[2] and psychiatry.[8] Interestingly, there is no specific UG curriculum for medicine even though the Royal College of Physicians (RCP), sets out postgraduate standards for specialty physicians in UK.

DISCUSSION

We used a structured literature search to identify publications which described core curriculum recommendations for a specialty or subject for UK medical undergraduates. We identified common factors and themes, and bodies who have developed a core curriculum for UK medical schools, grouping specialties into clinical, foundation and professionalism related subjects.

For peer review only - http://bmjopen.bmj.com/site/about/guidelines.xhtml

Medical schools have an enormous task of continuously responding to changes in clinical practice, keeping the curriculum in context and ensuring that students are not over burdened by content. From our review of the available literature, we demonstrated that most of the available specialty specific core-curricula have been developed through international and national expert consensus, advising on minimum standards on core knowledge and skills to be achieved by medical graduates and align themselves to the Outcomes for Graduates document. The 'core-knowledge' in specialty specific curricula could help develop minimum standards and provide quality assurance for both generic and specialty-specific areas across all UK medical schools. The drivers to curriculum development included patient safety, disease burden and needs of society and lack of representation or inadequate preparedness amongst graduates. We also identified areas where there are gaps or overlaps in some specialities' curricula. Though the GMC recommends a 'core curriculum' for UG medical education, medical educators have found it challenging to define what is meant by core, what needs to be included and its relevance to the bigger context of competency for graduating medical students.[1,2,3]

Whilst medical schools align their curricula to the generic Outcomes for Graduates, specialty representation and mapping of burden of disease in curricula is challenging, how individual providers of medical education map curriculum content and learning outcomes and assessments for specialties is not explicit.[41,42] As Voss et al have noted, 'Improving patient safety and quality in health care is one of medicine's most pressing challenges'.[43] The GMC has also highlighted these areas in the new Outcomes for Graduates.[3]

In the review, it was observed that there was duplication and overlapping of some specialties and subjects with their core-curricular recommendations. For the purpose of this review, curricula developed on topics within specialties were excluded. For example, a curriculum for delirium has been developed which is a topic covered within psychiatry and geriatrics specialty.[44] Similarly, recently, an Ultrasound curriculum[45] for medical students has been developed in addition to the national UG curriculum for radiology. Perhaps necessary, certain clinical conditions demand a multi-disciplinary approach. An integrated approach to combine specialties would

For peer review only - http://bmjopen.bmj.com/site/about/guidelines.xhtml

help reduce content overload, whilst keeping teaching and learning contextual. The introduction of longitudinal integrated clerkship in medical schools is looking to address this issue.[42] Further adoption of inter-professional education can help to change attitudes and behaviours and provide a sustainable model to accommodate large numbers of students.[46,47] For example, the non-surgical oncology and palliative care national curricula could be integrated and specialist nurses involved in helping medical students understand different aspects of cancer and end of life care.[11,12,26]

There are a number of drivers of curriculum development. For example, in 2016, the UK government announced strategies to fund, develop and grow the general practice workforce, to meet the increasing demands of an aging population with complex needs and chronic disease burden.[48] The new UG curriculum for general practice has been developed to complement postgraduate curriculum reflecting how curricula have evolved in relation to health care service and societal needs.[25] Furthermore, the introduction of a UK-wide Medical Licencing Assessment in 2022 may influence what medical schools teach and provide opportunities to keep curricular content relevant and inclusive.

Thus, by examining the evidence for need to change or update medical curricula, and through reflection on content and process, Mezirow's concept of transformative learning could be applied by medical educators in curricular development.[49] For instance in Scotland, the Scottish Deans' Medical Curriculum Group (SDMCG) discusses and helps coordinate the development, delivery and evaluation undergraduate curricula. Their aim is to ensure that graduates from each school in Scotland are of an equivalent standard. Their review of the curriculum has also been informed by frameworks on specialist subjects like acute and emergency medicine, dermatology, neurology, palliative care, pharmacology and sexual health.[50]

The curricula developed for foundation and professionalism related subjects recommended their components might be implemented by integration both horizontally and vertically throughout the medical course. These subjects, relevant and applicable to all clinical specialties, should be included in generic outcomes for the specialties and form a framework through the undergraduate years.

For peer review only - http://bmjopen.bmj.com/site/about/guidelines.xhtml

It is vital for UG curricula to evolve by keeping abreast with changing clinical needs and advances in diagnostics, investigative and management strategies.

With limited availability of resources and time, the curricula could be split into essential, desirable and nice to know categories to help prioritise areas of teaching and learning. The use of technological advances (e.g. use and sharing of e-learning resources/ webinars) could help provide flexibility and autonomy in how these curricula could be implemented and delivered, if followed.

LIMITATIONS OF THE REVIEW

We conducted a comprehensive search for all relevant articles for this study. However, as with limitations seen in scoping reviews, articles or studies pertinent to the review question may have been unintentionally omitted.[3] The level of evidence for the quality of the developed specialty specific core-curricula could not be graded in this search, as done with systematic reviews. However, most curricula were developed with expert consensus and use of a Delphi study, suggesting that efforts were made to establish 'best practice' and core curricular outcomes. The curricular components for individual specialties were variable, making comparisons difficult.

We were nevertheless able to establish common themes and drivers for development of the UG core-curricula for UK medical undergraduates. In addition, the study helped identify gaps in specialty specific curricula like the absence of an overarching RCP curricula, as well as the heterogeneity of the curricular designs between specialties.

CONCLUSIONS

This literature review highlights the current status of specialty specific core curricula for medical graduates in UK. This is the first study to provide a comprehensive overview of the curricula developed by specialties and their recommendations on knowledge and skills expected of all UK medical graduates, in alignment with GMC.

For peer review only - http://bmjopen.bmj.com/site/about/guidelines.xhtml

It would be interesting to see how medical schools are implementing and assessing these curricula and how that is likely to affect future training.

For peer review only

For peer review only - http://bmjopen.bmj.com/site/about/guidelines.xhtml

Contributorship statement:

All 3 authors have made substantial contributions to the planning, conduct, analyses and interpretation of findings, and reporting of the work described in the article, and have agreed to be accountable for all aspects of the work, its accuracy and integrity. MS is responsible for the overall content as guarantor.

MS, RM and GD with RP formulated the review question.

MS screened the titles and abstracts of retrieved citations and MS and RM assessed the full text. MS, RM and GD resolved any ambiguities about inclusion criteria. RM and MS devised the data extraction proforma. MS extracted data and discussed data items with RM and GD. MS contacted study authors for further information.

RM and MS identified common themes, MS conducted the narrative synthesis with RM and GD.

MS wrote the first draft of the manuscript and revised and amended it with RM and GD who also approved the final version to be published. MS is the corresponding author.

The corresponding author attests that all listed authors meet authorship criteria and that no others meeting the criteria have been omitted.

Funding: None

Conflicts of interest: None

Copyright/ Licence for publication:

The Corresponding Author has the right to grant on behalf of all authors and does grant on behalf of all authors, a worldwide licence to the Publishers and its licensees in perpetuity, in all forms, formats and media (whether known now or created in the future), to i) publish, reproduce, distribute, display and store the Contribution, ii) translate the Contribution into other languages, create adaptations, reprints, include within collections and create summaries, extracts and/or, abstracts of the Contribution, iii) create any other derivative work(s) based on the Contribution, iv) to exploit all subsidiary rights in the Contribution, v) the inclusion of electronic links from

For peer review only - http://bmjopen.bmj.com/site/about/guidelines.xhtml

the Contribution to third party material where-ever it may be located; and, vi) licence any third party to do any or all of the above."

Acknowledgements:

Dr Rakesh Patel, Clinical Associate Professor in Medical Education & Honorary Consultant Nephrologist, Faculty of Medicine & Health Sciences, University of Nottingham for guidance on the review question

Lisa Lawrence, Clinical librarian, Burton and Derby Teaching Hospitals NHS Foundation Trust, Derby for conducting the searches for the review

Prof Cathy Bennett, Royal College of Surgeons in Ireland (RCSI), Office of Research and Innovation. Manuscript revision.

Some of the work has been previously presented as oral presentation at:

British Association of Dermatologist (BAD) Teachers of Undergraduate Dermatology Annual Meeting on 6th March 2018

Association for the Study of Medical Education (ASME) Annual Scientific Meeting on 12th July 2018

Ethical approval: None required

Data availability statement: All data relevant to the study are included in the article and are from published sources cited within the article.

For peer review only

For peer review only - http://bmjopen.bmj.com/site/about/guidelines.xhtml

References:

1. Promoting excellence: standards for medical education and training. (2015). Manchester, pp.6, 7. Available at: https://www.gmc-uk.org/-/media/documents/Promoting_excellence_standards_for_medical_education_and_training_0715.pdf_61939165.pdf [Accessed 28 Sep. 2018].

2. Medical Licencing Assessment. (2018). General Medical Council. Available at: https://www.gmc-uk.org/education/standards-guidance-and-curricula/projects/medical-licensing-assessment [Accessed 28 Sep. 2018].

3. Outcomes for graduates. (2018).General Medical Council. Available at: https://www.gmc-uk.org/-/media/documents/dc11326-outcomes-for-graduates-2018_pdf-75040796.pdf [Accessed 28 Sep. 2018].

4. Future Hospital: caring for medical patients. (2013). Available at: https://www.rcplondon.ac.uk/projects/outputs/future-hospital-commission [Accessed 28 Sep. 2018].

5. Arksey H, O'Malley L. Scoping studies: towards a methodological framework. Int J Soc Res Methodol. 2005; 8 (1):19-32.

6. The Royal College of Paediatrics. Undergraduate Curriculum for child health. 2015. http://www.rcpch.ac.uk. [Accessed 3 Apr 2017].

7. Royal College of Obstetrics & Gynaecologists. National Undergraduate Curriculum in Obstetrics and Gynaecology.UK.2015. https://www.rcog.org.uk/en/careers-training/specialty-training-curriculum/undergraduate-training-in-og/ [Accessed 3 Apr 2017].

8. Royal College of Psychiatrists. Core Curriculum in Psychiatry. 2011. http://www.rcpsych.ac.uk/workinpsychiatry/faculties/academic/undergraduateteaching/curriculum.aspx. [Accessed 4 Apr 2017].

For peer review only - http://bmjopen.bmj.com/site/about/guidelines.xhtml

9. The Royal College of Surgeons. National Undergraduate Curriculum in Surgery.2015. https://www.rcseng.ac.uk//media/files/rcs/library-and-publications/non-journal-publications/undergraduate_curriculum_2015-web.pdf. [Accessed 14 May 2017].

10. Baldock C. The Acute Care Undergraduate TEaching (ACUTE) Initiative: consensus development of core competencies in acute care for undergraduates in the United Kingdom. Intensive Care Med. 2005; 31 (12):1627-33.

11. Linklater GT, Bowden J, Pope L et al. Developing learning outcomes for medical students and foundation doctors in palliative care: a national consensus-seeking initiative in Scotland. Med Teach. 2014; 36 (5): 441-446.

12. Paes P, Wee B. A Delphi study to develop the Association for Palliative Medicine consensus syllabus for undergraduate palliative medicine in Great Britain and Ireland. Palliative medicine. 2008; 22 (4): 360-364.

13. Association of British Neurologists. Undergraduate Neurology.2017. http://www.theabn.org/what-we-do/training/undergraduate-neurology.html [Accessed 14 May 2017].

14. British Association of Dermatologists. Undergraduate Dermatology Curriculum.2016 http://www.bad.org.uk/librarymedia/documents/BAD%202016%20revised%20UG%20Curriculum%20(UK).pdf. [Accessed 14 May 2017].

15. Smith CF, Finn GM, Stewart J, McHanwell S. Anatomical Society core regional anatomy syllabus for undergraduate medicine: The Delphi process. J.Anat. 2016; 228 (1):2-14.

16. The Royal College of Pathologists. Pathology Undergraduate Curriculum. 2014. https://www.rcpath.org/resourceLibrary/pathology-undergraduate-curriculum-.html. [Accessed 3 Apr 2017].

For peer review only - http://bmjopen.bmj.com/site/about/guidelines.xhtml

17. Jackson, A.A., Human nutrition in medical practice: the training of doctors. Proc Nut Soc. 2001; 60 (2): 257-63.

18. Stirrat GM, Johnston C, Gillon R. Boyd K. Medical ethics and law for doctors of tomorrow: the 1998 Consensus Statement updated. J med ethics. 2010; 36 (1):55-60.

19. Masud T, Blundell A, Gordon AL, et al. European undergraduate curriculum in geriatric medicine developed using an international modified Delphi technique. Age and ageing. 2014; 43 (5):695-702.

20. Bachmann C, Abramovitch H, Barbu CG, et al. A European consensus on learning objectives for a core communication curriculum in health care professions. Patient Educ Couns. 2013; 93 (1):18-26.

21. Noble LM, Scott-Smith W, O'Neill B, Salisbury H. Consensus statement on an updated core communication curriculum for UK undergraduate medical education. 2018; 101 (9) 1712-19.

22. British Geriatrics Society Recommended Curriculum in Geriatric Medicine for MedicalUndergraduates.2013. http://www.bgs.org.uk/generalinformation171/secttrainees/generalinfo/undergraduatecurriculum. [Accessed 14 May 2017].

23. Woolf AD, Walsh NE. Global core recommendations for a musculoskeletal undergraduate curriculum. Ann Rheum Dis. 2004; 63:517-24.

24. Coady D, Walker D, Kay L. Regional Examination of the Musculoskeletal System (REMS): a core set of clinical skills for medical students. Rheumatology. 2004; 43: 633–639.

25. Royal College of General Practitioners. (2018).Teaching general practice. Guiding principles for undergraduate general practice curricula in UK medical schools. [Accessed 3 March 2019].

26. The Royal College of Radiologists. Medical undergraduate non-surgical oncologycurriculum.2014. https://www.rcr.ac.uk/clinical-oncology/undergradute-oncology. [Accessed 4 May 2017].

For peer review only - http://bmjopen.bmj.com/site/about/guidelines.xhtml

27. The Royal College of Radiologists. Undergraduate radiology curriculum second edition. London, 2017. www.rcr.ac.uk. [Accessed 4 Apr 2017].

28. Royal College of Ophthalmologists. "Eyes & Vision Curriculum" for Undergraduate and Foundation Doctors. 2015. https://www.rcophth.ac.uk/wp-content/uploads/2014/07/Undergraduate-and-Foundation-doctors-curriculum.pdf.[Accessed 3 Apr 2017].

29. Public Health Educators in Medical Schools. Undergraduate Public Health Curriculum for UK Medical Schools. Consensus Statement 2014.http://www.fph.org.uk/uploads/PHEMS booklet web.pdf. [Accessed 14 May 2017].

30. Ross S, Maxwell S. Prescribing and the core curriculum for tomorrow's doctors: BPS curriculum in clinical pharmacology and prescribing for medical students. Br. J. Clin. Pharmacol. 2012; 74 (4):644-61.

31. Core curriculum for sociology in undergraduate medical education. Behavioural & Social Sciences Teaching in Medicine (BeSST) Sociology SteeringGroup.2016.http://www.ed.ac.uk/usher/news-events/news-2016/core-curriculum-sociology-medical-education. [Accessed 20 Apr 2017].

32. Turner K, Brewster S. Undergraduate urology: A survey of current provisions and guidelines for a core curriculum. BJU Int. 2002; 90 (4): 475.

33. The British Association of Urological Surgeons. An undergraduate syllabus for urology.2013. https://www.baus.org.uk/_userfiles/pages/files/professionals/education/Undergraduate%20Syllabus.pdf. [Accessed 14 May 2017].

34. Lyoyd S, Tan ZE, Taube M-A, Doshi J. Development of an ENT undergraduate curriculum using a Delphi survey. Clin Otolaryngol. 2014; 39:281-288.

35. Bleakley A, Marshall R, Brömer R. Toward an aesthetic medicine: developing a core medical humanities undergraduate curriculum. J med humanit. 2006; 27 (4):197-213.

For peer review only - http://bmjopen.bmj.com/site/about/guidelines.xhtml

36. WHO Multi-professional Patient Safety Curriculum Guide.2011. World Health Organization.http://www.who.int/patientsafety/education/mp_curriculum_guide/en/ [Accessed 3 Apr 2017].

37. Burge SM. Undergraduate medical curricula: are students being trained to meet future service needs? Clin Med.2003; 3 (3): 243-6.

38. Association of British Neurologists Training & Education Subcommittee. Clinical Neurology Medical Undergraduate Curriculum UK Medical School Survey.2002. http://www.theabn.org/media/Med%20Sch%20Neuro%20Survey%202002-3.pdf [Accessed 30/7/14] and CM Wiles, P Bain (Personal communications 2011).

39. Wilkinson IMS. A survey of undergraduate teaching of clinical neurology in the UK 1990. J Neurol Neurosurg Psych. 1991; 54:266-68.

40. General Medical Council (2010) Standards for curricula and assessment systems. 2010. http://www.gmc-uk.org/education/postgraduate/standards_forcurricula_and_assessment_systems.asp. [Accessed 14 May 2017].

41. McIntosh N, Olver RE, Haddad, D. What is core? Guidelines for the core curriculum in paediatrics. Med Educ.1997; 31(5): 354-8.

42. Hirsh D, Gaufberg E, Ogur B, Cohen P, Krupat E, Cox M, Pelletier S, Bor D. Educational outcomes of the Harvard Medical School-Cambridge integrated clerkship: a way forward for medical education. Acad Med. 2012. 87(5): 643-50.

43. Voss JD, May NB, Schorling JB et al. Changing conversations: teaching safety and quality in residency training. Academic Medicine.2008; 83:1080-7.

44. Copeland C, Fisher J, Teodorczuk A. Development of an international undergraduate curriculum for delirium using a modified delphi process. Age and Ageing. 2018; 47. 131-73.

For peer review only - http://bmjopen.bmj.com/site/about/guidelines.xhtml

45. Wakefield RJ, Weerasingheb A Tung P, et al. The development of a pragmatic, clinically driven ultrasound curriculum in a UK medical school Medical Teach 2018; 40(6): 600–606.

46. Ponzer S, Hylin U, Kusuoffsky A et al. Interprofessional training in the context of clinical practice: goals and students' perceptions on clinical education wards. Medical Education. 2004; 38(7):727-36.

47. Anderson ES, Lennox A. The Leicester Model of Interprofessional education: developing, delivering and learning from student voices for 10 years. J Interprof Care.2009; 23(6):557-73.

48. NHS England. General Practice Forward View.2016. https://www.england.nhs.uk/gp/gpfv/ [Accessed 22 May 2017].

49. Mezirow J and associates (eds). Fostering Critical Reflection in Adulthood. 1990. Jossey-Bass, San Francisco, CA.

50. Scottish Deans' Medical Curriculum Group. The Scottish Doctor: Learning Outcomes for the medical undergraduate in Scotland: a Foundation for Competent and Reflective Practitioners. 2008: Dundee. UK. http://www.scottishdoctor.org/resources/scotdoc3.pdf[Accessed 22 May 2017]

For peer review only

For peer review only - http://bmjopen.bmj.com/site/about/guidelines.xhtml

Table 1: Electronic data collection table of specialty or subject with curriculum in alphabetical order

	Specialty with curriculum	Year	Organization/Body	Alignment with GMC outcomes
**1.**	**Acute care**[10]	2005	Resuscitation Council UK	Y
**2.**	**Clinical pharmacology and prescribing**[30]	2012	British Pharmaceutical Society	Y
**3. a**	**Core communications curriculum**[20]	2013	European study using Delphi process	
**b**	**Communications curricula**[21]	2018	U. K. Council for Clinical Communication Skills Teaching in Undergraduate Medical Education	Y
**4.**	**Dermatology**[14]	2016	British association of dermatologists	Y
**5.**	**ENT**[34]	2014	A Delphi survey (UK) of ENT consultants and specialist registrars, accident and emergency consultants and specialist registrars,	Y

For peer review only - http://bmjopen.bmj.com/site/about/guidelines.xhtml

			general practitioners and paediatricians.	
**6.**	**General Practice** [25]	2018	Royal college of general practitioners	Y
**7. a**	European undergraduate curriculum in **geriatric medicine**[19]	2014	European Union of Medical Specialists	Y
b	**Geriatric medicine** for undergraduates[21]	2013	British Geriatrics Society	Y
**8.**	**Medical Humanities**[35]	2006	Peninsula medical school	Unclear
**9. a**	**Musculoskeletal system (MSK)**[23]	2004	The Bone and Joint Decade (BJD) Undergraduate Curriculum Development Group	Y
b	**Regional Examination of the Musculoskeletal System (REMS)**[24]	2004	UK representation of the four specialties- Rheumatologists, Orthopaedic Surgeons, Geriatricians and General Practitioners	Y
**10.**	**Neurology**[13]	2017	Association of British Neurologists	Y

For peer review only - http://bmjopen.bmj.com/site/about/guidelines.xhtml

**11.**	Human **Nutrition**[17]	2001	National Nutrition Task Force	Y
**12..**	**Obstetrics & Gynaecology**[7]	2015	Royal College of Obstetricians and Gynaecologists (RCOG)	Y
**13.**	**Ophthalmology**[28]	2015	Royal college of Ophthalmologists	Y
**14.**	**Oncology**[26]	2014	The Royal College of Radiologists	Y
**15.**	**Paediatrics**[6]	2015	Royal college of paediatrics and child health	Y
**16. a**	**Palliative Care**[11]	2014	Scottish Palliative Medicine Curriculum Development Group	Y
**b**	**Palliative Medicine**[12]	2008	Association for Palliative Medicine	Y
**17.**	**Pathology**[16]	2014	The Royal College of Pathologists	Y
**18..**	**Patient Safety**[36]	2011	World Health Organization	Y
**19.**	**Psychiatry**[8]	2011	Royal college of psychiatrists	Y
**20.**	**Public Health**[29]	2014	Faculty of Public Health	Y
**21.**	**Radiology**[27]		Royal college of	Y

For peer review only - http://bmjopen.bmj.com/site/about/guidelines.xhtml

		2017	radiologists	
**22.**	**Sociology**[31]	2016	Behavioural & Social Sciences Teaching in Medicine (BeSST) Sociology Steering Group	Y
**23.**	**Surgery**[9]	2015	Royal college of Surgeons	Y
**26. a**	**Urology**[32]	2002	Journal article in Urology	Unclear
**b**	**Urology syllabus**[33]	2012	British Association of Urological Surgeons	

For peer review only - http://bmjopen.bmj.com/site/about/guidelines.xhtml

Figure 1: PRISMA (preferred reporting items for systematic reviews and meta-analysis) flow diagram of the scoping review

UG= undergraduate

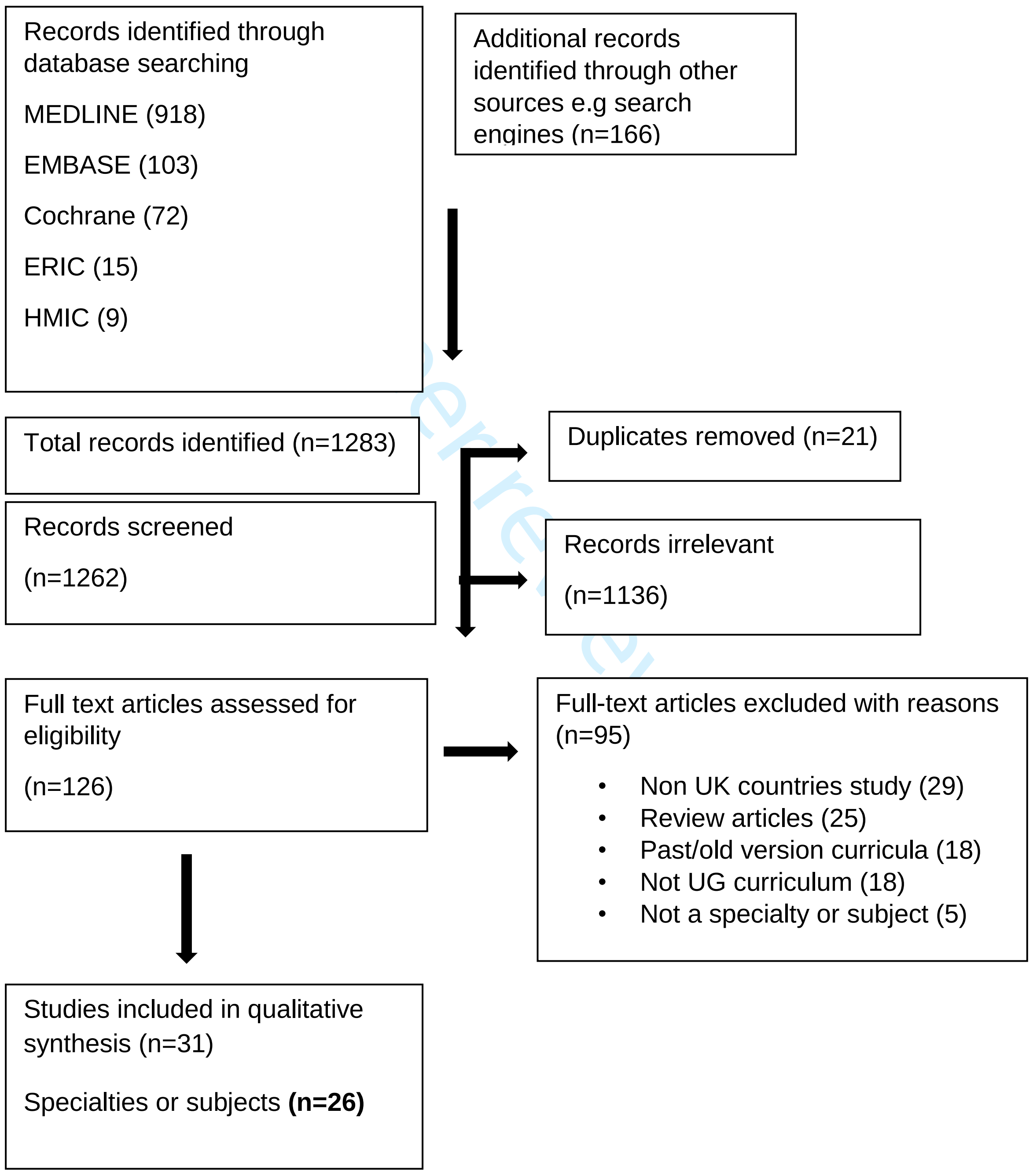

Example Search Strategy for Appendix

NICE Healthcare Databases Advanced Search MEDLINE 1946 to present (Provided by Proquest).

Strategy 606888

#	Database	Search term	Results
1	Medline	"EDUCATION, MEDICAL, UNDERGRADUATE"/	22398
2	Medline	("medical education").ti,ab	36006
3	Medline	(undergraduate).ti,ab	29224
4	Medline	("medical student").ti,ab	6528
5	Medline	"STUDENTS, MEDICAL"/	30311
6	Medline	(2 AND 3)	3401
7	Medline	(1 OR 4 OR 5 OR 6)	48667
8	Medline	CURRICULUM/	70249
9	Medline	(curricul*).ti,ab	48931
10	Medline	(8 OR 9)	93842
11	Medline	(core OR influenc* OR design* OR develop* OR deliver* OR enabl* OR barrier* OR process* OR factor*).ti,ab	9645339
12	Medline	"REFERENCE STANDARDS"/	40365
13	Medline	(standard*).ti,ab	1110534
14	Medline	(12 OR 13)	1133896
15	Medline	(11 OR 14)	10194258
16	Medline	DERMATOLOGY/	17224

For peer review only

17	Medline	(dermatology).ti,ab	23551
18	Medline	(16 OR 17)	33537
19	Medline	exp "UNITED KINGDOM"/	350174
20	Medline	(UK OR "United Kingdom" OR "Great Britain" OR GB OR England OR Wales OR "Northern Ireland" OR Scotland).ti,ab	205051
21	Medline	(19 OR 20)	459845
22	Medline	(7 AND 10 AND 15)	8130
23	Medline	22 [DT 1994-2019]	7323
24	Medline	(7 AND 10 AND 15 AND 18)	57
25	Medline	24 [DT 1994-2019]	52
26	Medline	(7 AND 10 AND 15 AND 21)	788
27	Medline	26 [DT 1994-2019]	718
28	Medline	(7 AND 10 AND 15 AND 18 AND 21)	8
29	Medline	28 [DT 1994-2019]	8

For peer review only

Preferred Reporting Items for Systematic reviews and Meta-Analyses extension for Scoping Reviews (PRISMA-ScR) Checklist

SECTION	ITEM	PRISMA-ScR CHECKLIST ITEM	REPORTED ON PAGE #
**TITLE**			
Title	1	Identify the report as a scoping review.	1
**ABSTRACT**			
Structured summary	2	Provide a structured summary that includes (as applicable): background, objectives, eligibility criteria, sources of evidence, charting methods, results, and conclusions that relate to the review questions and objectives.	2
**INTRODUCTION**			
Rationale	3	Describe the rationale for the review in the context of what is already known. Explain why the review questions/objectives lend themselves to a scoping review approach.	4
Objectives	4	Provide an explicit statement of the questions and objectives being addressed with reference to their key elements (e.g., population or participants, concepts, and context) or other relevant key elements used to conceptualize the review questions and/or objectives.	4
**METHODS**			
Protocol and registration	5	Indicate whether a review protocol exists; state if and where it can be accessed (e.g., a Web address); and if available, provide registration information, including the registration number.	Not applicable
Eligibility criteria	6	Specify characteristics of the sources of evidence used as eligibility criteria (e.g., years considered, language, and publication status), and provide a rationale.	5 and 6
Information sources*	7	Describe all information sources in the search (e.g., databases with dates of coverage and contact with authors to identify additional sources), as well as the date the most recent search was executed.	5 and appendix 1
Search	8	Present the full electronic search strategy for at least 1 database, including any limits used, such that it could be repeated.	5 and appendix 1
Selection of sources of evidence†	9	State the process for selecting sources of evidence (i.e., screening and eligibility) included in the scoping review.	5,6
Data charting process‡	10	Describe the methods of charting data from the included sources of evidence (e.g., calibrated forms or forms that have been tested by the team before their use, and whether data charting was done independently or in duplicate) and any processes for obtaining and confirming data from investigators.	6 and Data from individual studies extracted into Table 1
Data items	11	List and define all variables for which data were sought and any assumptions and simplifications made.	6 and Table 1

St. Michael's
Inspired Care.
Inspiring Science.

For peer review only - http://bmjopen.bmj.com/site/about/guidelines.xhtml

SECTION	ITEM	PRISMA-ScR CHECKLIST ITEM	REPORTED ON PAGE #
Critical appraisal of individual sources of evidence§	12	If done, provide a rationale for conducting a critical appraisal of included sources of evidence; describe the methods used and how this information was used in any data synthesis (if appropriate).	Study quality appraisal not applicable.
Synthesis of results	13	Describe the methods of handling and summarizing the data that were charted.	Table 1
**RESULTS**			
Selection of sources of evidence	14	Give numbers of sources of evidence screened, assessed for eligibility, and included in the review, with reasons for exclusions at each stage, ideally using a flow diagram.	5 and PRISMA diagram
Characteristics of sources of evidence	15	For each source of evidence, present characteristics for which data were charted and provide the citations.	Table 1 and page 7
Critical appraisal within sources of evidence	16	If done, present data on critical appraisal of included sources of evidence (see item 12).	Not applicable
Results of individual sources of evidence	17	For each included source of evidence, present the relevant data that were charted that relate to the review questions and objectives.	7-10 (results section)
Synthesis of results	18	Summarize and/or present the charting results as they relate to the review questions and objectives.	7-10 Narrative synthesis of results
**DISCUSSION**			
Summary of evidence	19	Summarize the main results (including an overview of concepts, themes, and types of evidence available), link to the review questions and objectives, and consider the relevance to key groups.	10-13
Limitations	20	Discuss the limitations of the scoping review process.	13
Conclusions	21	Provide a general interpretation of the results with respect to the review questions and objectives, as well as potential implications and/or next steps.	13
**FUNDING**			
Funding	22	Describe sources of funding for the included sources of evidence, as well as sources of funding for the scoping review. Describe the role of the funders of the scoping review.	14

JBI = Joanna Briggs Institute; PRISMA-ScR = Preferred Reporting Items for Systematic reviews and Meta-Analyses extension for Scoping Reviews.

* Where *sources of evidence* (see second footnote) are compiled from, such as bibliographic databases, social media platforms, and Web sites.

† A more inclusive/heterogeneous term used to account for the different types of evidence or data sources (e.g., quantitative and/or qualitative research, expert opinion, and policy documents) that may be eligible in a scoping review as opposed to only studies. This is not to be confused with *information sources* (see first footnote).

‡ The frameworks by Arksey and O'Malley (6) and Levac and colleagues (7) and the JBI guidance (4, 5) refer to the process of data extraction in a scoping review as data charting.

§ The process of systematically examining research evidence to assess its validity, results, and relevance before using it to inform a decision. This term is used for items 12 and 19 instead of "risk of bias" (which is more applicable to systematic reviews of interventions) to include and acknowledge the various sources of evidence that may be used in a scoping review (e.g., quantitative and/or qualitative research, expert opinion, and policy document).

For peer review only - http://bmjopen.bmj.com/site/about/guidelines.xhtml

BMJ Open

DO WE NEED A CORE-CURRICULUM FOR MEDICAL STUDENTS? A SCOPING REVIEW

Journal:	*BMJ Open*
Manuscript ID	bmjopen-2018-027369.R2
Article Type:	Research
Date Submitted by the Author:	28-Jun-2019
Complete List of Authors:	Sharma, Maulina; University of Nottingham, Medical Education Centre, School of Medicine; University Hospitals of Derby & Burton NHS Foundation Trust , Dermatology<br>Murphy, Ruth; University of Nottingham, School of Medicine<br>Doody, Gillian; University of Nottingham, Medical Education Centre, School of Medicine
**Primary Subject Heading**:	Medical education and training
Secondary Subject Heading:	Medical education and training
Keywords:	Undergraduate, MEDICAL EDUCATION & TRAINING, United Kingdom, Core, Curriculum

SCHOLARONE™
Manuscripts

DO WE NEED A CORE-CURRICULUM FOR MEDICAL STUDENTS? A SCOPING REVIEW

Maulina Sharma, Ruth Murphy, Gillian A Doody

Medical Education Unit, University of Nottingham, School of Medicine, Nottingham, NG7 2UH, UK

Maulina Sharma, Consultant Dermatologist, Honorary Clinical Associate Professor, University of Nottingham, School of Medicine, Department of Dermatology London Road Community Hospital, University Hospitals of Derby & Burton NHS Foundation Trust, Derby DE1 2QY, UK

Ruth Murphy, Consultant Dermatologist, University of Nottingham, School of Medicine, Nottingham, NG7 2UH, UK

Gillian A Doody, Dean of Medical Education, Medical Education Unit, University of Nottingham, School of Medicine, Nottingham, NG7 2UH, UK

Corresponding author:

M Sharma email : msxms16@nottingham.ac.uk

Tel no: 01332 254653

Word count: 3020

Keywords: core curriculum, medical undergraduates, UK

For peer review only - http://bmjopen.bmj.com/site/about/guidelines.xhtml

ABSTRACT

Objective

The General Medical Council (GMC) recommends medical schools to develop and implement curricula that enable students to achieve learning outcomes required as graduates. UK medical schools follow the GMC's Outcomes for Graduates which can be viewed as generic. There are also plans for the GMC to introduce a national Medical Licencing Assessment (MLA) for medical graduates who want to practice medicine in the UK in 2022. With no standardised or unified undergraduate (UG) curriculum in UK, various specialities have expressed concerns about not being represented in medical schools and have developed specialty-specific core-curricula.

The aim of the scoping review was to identify learned bodies who have developed a core-curriculum for UK medical schools and highlight the drivers, gaps and future approaches to curricular development and implementation.

Methods

A literature search was conducted using online databases (EMBASE, MEDLINE, ERIC, HMIC, PubMed and CDSR) and online search engines (Google & Google Scholar, Department of Health, GMC and BMA) for relevant articles from 1996 to May 2017 (i.e. past 20 years) and the search updated for relevant articles up to 5th March 2019. A methodological framework to map the key concepts of undergraduate medical curriculum was followed. Any national body relevant to UK medical undergraduates with a core-curriculum was included.

Results

A total of 1283 articles were identified, 31 articles were eligible for inclusion, detailing undergraduate curricula for 26 specialties: 18 clinical specialties, 4 foundation subjects, 4 based on professionalism

European, national and regional expert bodies developed these curricula and provided guidance to medical schools on minimum standards for knowledge and skills required.

Conclusions

For peer review only - http://bmjopen.bmj.com/site/about/guidelines.xhtml

This study identifies core curricula developed and recommended for UK medical students. It explores common themes, drivers and gaps in UG curricula development and the need for UK medical schools to align and revise their curricula and standards.

Strengths and limitations of the study

- Scoping review of literature as the methodological approach in the study helped identify, categorise and provide a narrative synthesis of the key aspects on core curricula for UK medical students.
- Heterogeneity in the curricular designs for specialties made comparisons between them difficult.
- As with limitations seen in other scoping reviews, articles or studies pertinent to the review question may have been unintentionally omitted.

For peer review only

For peer review only - http://bmjopen.bmj.com/site/about/guidelines.xhtml

INTRODUCTION

The General Medical Council (GMC) in 2016, set out the Promoting Excellence document which outlines a set of standards for all stages of medical education and training, including undergraduate (UG) medical education.[1] These standards should ensure that that medical schools develop and implement curricula to enable students to achieve learning outcomes required as graduates and practise safely and competently. The GMC also plans to introduce a Medical Licencing Assessment (MLA) in 2022 applicable to medical graduates who want to practice medicine in the UK.[2] Specialities have expressed concern about medical curricula as medical schools in the UK currently organise their own curricula and assessments based on GMC's Outcomes for Graduates which can be viewed as generic.[3] This may result in scanty representation of specialist knowledge and skills within the curriculum. Once the students successfully graduate, the GMC grants them licence to practice.

In the UK there is a growing need for development of medical generalists, that is, those who treat all common medical conditions and refer patients into secondary care for urgent or specialist treatment. A 'Future Hospital Commission' report (FHC), through the Royal College of Physicians (RCP) in 2013 promoted the re-introduction of generalism into medical training.[4] This means that medical education and training of doctors in UK needs to address the current and future demographic of patients.

With the generalist trend, as well as plans to introduce the MLA for all medical graduates in the UK, medical schools may be influenced on what they teach and assess. There is currently no unified or standardized UG medical curriculum in the UK. Various specialities have expressed concerns about not being adequately in medical school curricula and have developed specialty-specific core-curricula.

Our objective was to perform a scoping review of literature produced by learned bodies who have developed a core-curriculum for UK medical schools and to use this information to highlight the drivers, gaps and future approaches to curricular development and implementation.

For peer review only - http://bmjopen.bmj.com/site/about/guidelines.xhtml

METHODS

We performed a scoping review using the methodological framework as outlined by Arksey and O'Malley.[5] The framework was used to; identify the specialties, or subjects, which recommended and had developed an undergraduate core-curriculum for UK medical schools; select and search for relevant studies; chart and collate the data and summarise the results.

Search strategy

A literature search was conducted using online databases (EMBASE, MEDLINE, HMIC (searched via NICE Healthcare Databases Advanced Search), PubMed, The Cochrane Library and ERIC (Education Resources Information Centre). Other online search engines (Google & Google Scholar) were then searched as well as the Department of Health, GMC and BMA websites for relevant articles from 1996 to May 2017 (i.e. the past 20 years). The literature search was updated till 5th March 2019 for any further recent relevant articles.

Keywords or terms used in the searched included: General medical council (GMC), medical student, medical education, undergraduate, curriculum, standards and national (UK and individual countries e.g. England). An example of search strategy is included in Appendix 1.

Electronic search results were managed using a reference manager. Titles and abstracts of all citations were first screened by the author (MS), those that were not related to undergraduate curricula or the UK were excluded. Full articles were requested from the library, when it was not possible to eliminate them from reading either their title or abstract. The full text of all potentially eligible articles was then obtained and assessed against the inclusion criteria (MS and RM). Where the full text of an article was not available the authors were contacted to request a copy. Any ambiguities about whether a study met the inclusion criteria were resolved by a consensus between authors (MS, RM, and GD).

Eligibility criteria

For peer review only

For peer review only - http://bmjopen.bmj.com/site/about/guidelines.xhtml

Inclusion criteria were all published studies, reports or articles which had a recommended core-curriculum for a specialty or subject for UK medical undergraduates on a national level were eligible. Any national body, society, college or organisation specific and pertinent to UK medical undergraduate education was included. For curricula which had been revised or updated, the most recent (up to 05/03/19) were included.

Exclusion criteria were articles pertaining to core-curricula for postgraduate courses and those not specific to UK medical undergraduates. Due to the study being relevant to UK setting, non-English language literature was excluded.

Patient and Public involvement: As this was a scoping review of available undergraduate core-curricula, patients or public were not involved.

Data management

Data were collated and reported using PRISMA flow diagram (Figure 1). Characteristics of each study were recorded in an electronic data collection table and included the name of specialty, or subject, in alphabetical order, organisational body for developing the curricula, year of curriculum update, and whether studies were aligned to GMC learning outcomes were then extracted and recorded on an electronic data collection table (Table 1).

The key concepts derived were broadly grouped into the following: categories of core-curricula and their timing within the school course; organisations involved in development of the core-curricula; drivers for development of core-curricula; and curriculum overlaps and gaps.

RESULTS

The initial search identified a total of 1283 articles, after duplicates were removed (n=21). All articles were screened and 126 were initially highlighted as of interest. Of these, 95 were subsequently excluded, with reasons recorded. A total of 31 articles, published since 2002, and describing core curricula recommendations for medical undergraduates were then included in the narrative synthesis. These comprised a

For peer review only - http://bmjopen.bmj.com/site/about/guidelines.xhtml

total of 26 specialties or subjects (Figure 1). Of these 26 specialties, 5 specialties had additional relevant articles (Table1).

Narrative synthesis of results

Having identified publications from learned bodies who have developed a core-curriculum for UK medical schools, we identified the following themes and drivers for curricular development and implementation and summarised areas where there are gaps and overlaps which may influence future approaches to curricular development and implementation.

i) Grouping of curricula and timing within the school course

The curricula developed and recommended for UK medical schools could be grouped into 3 main categories: clinical specialty based (n=18) (e.g. surgery, dermatology); foundation subjects (n=4) (e.g. anatomy, pharmacology) and subjects related to professionalism (n=4) (e.g. communication skills) (Table 1).

Clinical specialty curricula were applicable during clinical years of training e.g. paediatrics, obstetrics & gynaecology, psychiatry, surgery, acute medical care, resuscitation and end of life care.[6-12] Some specialties had developed a curricula with a system-based approach e.g. neurology and dermatology.[13,14] Foundation subjects like anatomy, pathology and nutrition as well as curricula related to professionalism, like medical ethics and law, were developed so as to be relevant throughout all medical school training.[15-18]

ii) Organisations involved in development of individual core-curricula

Curricula were developed by international, national and regional organisations with participation from relevant stakeholders. Consensus agreement derived from a Delphi process was the preferred method of achieving core-curricular recommendations.[10, 11, 15, 18,-21,]

For peer review only
For peer review only - http://bmjopen.bmj.com/site/about/guidelines.xhtml

Four curricula, namely communications skills, geriatrics, musculoskeletal system and palliative care had their core-curriculum co-developed by two different international and national bodies. The international recommendations encompassed the national guidance.[19-24]

The Royal Colleges of UK developed specialty specific core-curricula for 9 specialties (e.g. general practice, pathology, radiology, surgery) [6-9, 16, 25-28]

National specialist consultant societies and bodies developed 14 specialty specific curricula (e.g. dermatology, medical ethics, palliative care) [10—15, 17, 18, 21-24, 29, 30, 31].

National consultant expert consensus was used to provide guidelines for a core curriculum in urology and ENT, which was developed through a survey of consultants whilst a separate urology syllabus for medical undergraduates was developed by the British Association of Urological Surgeons.[32-34] At a regional level, one medical school developed and recommended a curriculum for medical humanities.[35]

i) Drivers for development of core-curricula

For peer review only

For peer review only - http://bmjopen.bmj.com/site/about/guidelines.xhtml

a) *Patient safety*

Patient safety was a common theme in the development of core-curricula for clinical specialties, foundation subjects, in addition to patient expectations of a graduate in terms of medical ethics and law. [15,18,21,27,22,23] The World Health Organisation (WHO), have published the multi-professional patient safety curriculum guide to aid teaching of patient safety in universities and schools, including medicine.[36] The ACUTE initiative identified the core set of competencies for resuscitation and care of acutely ill patients that all undergraduate trainees require to be competent in at the point of graduation.[10] This was designed to address the sub-optimal care of the critically ill in hospital where contributory factors included recognition of critical illness, lack of knowledge, a lack of supervision, failure to seek advice and poor communication.[10] Similarly, the British Pharmacology Society (BPS) prepared a curriculum to standardise the learning objectives and recommended introduction of prescribing safety assessments in all medical schools, in alignment with GMC's outcomes.[30,]

b) *Disease burden and needs of society*

In the UK, cancer affects 1 in 3 people.[26] The Royal College of Radiologists who developed the UG radiology curriculum also developed the non-surgical oncology curriculum. With collaboration from the Royal College of Physicians' Joint Collegiate Council for Oncology (JCCO), the UG curriculum with its joint approach aims to address competencies in a multidisciplinary setting towards patients affected with cancer and their needs.[26, 27]

Societal requirements of an ageing, frail population, with complex problems related to independence and mobility were important factors for the development of a core-curriculum for geriatric medicine and musculoskeletal system. [22-24]

The National Health Service of UK is underpinned by General Practitioners (GP) in UK, who remain the first point of contact for patients and provide long-term continuity of care. To provide a sustainable service to patients, UK needs approximately half of medical school graduates to pursue a career in family medicine or general practice.[25]

For peer review only - http://bmjopen.bmj.com/site/about/guidelines.xhtml

In 2018, the Royal College of General Practitioners (RCGP) with Society for Academic Primary Care (SAPC) collaborated to develop the GP curriculum as a guidance to UK medical schools on the design and delivery of teaching of general practice to help bridge the gap between societal demands and needs. [25]

Public healthcare prevention and promotion, human nutrition and its relevance to health and disease also had specific UG curricula.[17, 25]

c) Lack of representation and inadequate preparedness amongst graduates

Multiple specialties, through national surveys across UK medical schools, felt a lack of adequate representation, teaching or assessments in medical school curricula.[13,14,32-34,37] These concerns were echoed by feelings of inadequacy amongst junior doctors and general practitioners due to a lack of training during undergraduate training in some specialties, some of these specialties have subsequently developed their own curricula.[13,38,39] This is particularly seen in specific core-curricula like ENT and Dermatology, where related diseases and conditions are common presentations in UK primary care.[14,34] This has led such specialties to develop specialty specific learning objectives, defining minimum knowledge and skills to be attained.

iv) Curriculum: overlaps and gaps

The General Medical Council, based on a review of curriculum theories, its relevance and context with regards to medical education and its stakeholders i.e. students, teachers and regulators, defines "a curriculum as a statement of the intended outcomes, encompassing content, teaching, learning and assessment methods, feedback and supervision as part of the educational programme".[40]

This scoping review found that the Royal Colleges of UK, like the Royal College of Surgeons (RCS) and Obstetrics and Gynaecology (RCOG) covered the curricular requirements comprehensively by highlighting content and how to achieve the learning objectives with structure and assessment methods.[7, 9]

For peer review only - http://bmjopen.bmj.com/site/about/guidelines.xhtml

However, many clinical and foundation subjects suggested medical schools determine how best to incorporate content within their medical course to allow flexibility in their implementation, delivery and assessment. Hence, they were focussed primarily on syllabus content. [13,14,28,34]

Despite representation of surgical sub-specialties like ENT and urology in the RCS curriculum, both sub-specialities developed specific outcomes for medical school curricula.[32-4] Overlapping of core-content was also seen between more established curricula like geriatrics[2] and psychiatry.[8] Interestingly, there is no specific UG curriculum for medicine even though the Royal College of Physicians (RCP), sets out postgraduate standards for specialty physicians in UK.

DISCUSSION

We used a structured literature search to identify publications which described core curriculum recommendations for a specialty or subject for UK medical undergraduates. We identified common factors and themes, and bodies who have developed a core curriculum for UK medical schools, grouping specialties into clinical, foundation and professionalism related subjects.

Medical schools have an enormous task of continuously responding to changes in clinical practice, keeping the curriculum in context and ensuring that students are not overburdened by content. From our review of the available literature, we demonstrated that most of the available specialty specific core-curricula have been developed through international and national expert consensus, advising on minimum standards on core knowledge and skills to be achieved by medical graduates and align themselves to the Outcomes for Graduates document. The 'core-knowledge' in specialty specific curricula could help develop minimum standards and provide quality assurance for both generic and specialty-specific areas across all UK medical schools. The drivers to curriculum development included patient safety, disease burden and needs of society and lack of representation or inadequate preparedness amongst graduates. We also identified areas where there are gaps or overlaps in some specialities' curricula. Though the

For peer review only - http://bmjopen.bmj.com/site/about/guidelines.xhtml

GMC recommends a ‘core curriculum’ for UG medical education, medical educators have found it challenging to define what is meant by core, what needs to be included and its relevance to the bigger context of competency for graduating medical students.[1,2,3]

Whilst medical schools align their curricula to the generic ‘Outcomes for Graduates’, specialty representation and mapping of burden of disease in curricula is challenging, how individual providers of medical education map curriculum content and learning outcomes and assessments for specialties is not explicit.[41,42] As Voss et al have noted, ‘Improving patient safety and quality in health care is one of medicine’s most pressing challenges’.[43] The GMC has also highlighted these areas in the new Outcomes for Graduates.[3]

In the review, it was observed that there was duplication and overlapping of some specialties and subjects with their core-curricular recommendations. For this review, curricula developed on topics within specialties were excluded. For example, a curriculum for delirium has been developed which is a topic covered within psychiatry and geriatrics specialty.[44] Similarly, recently, an Ultrasound curriculum[45] for medical students has been developed in addition to the national UG curriculum for radiology. Perhaps necessary, certain clinical conditions demand a multi-disciplinary approach. An integrated approach to combine specialties would help reduce content overload, whilst keeping teaching and learning contextual. The introduction of longitudinal integrated clerkship in medical schools is looking to address this issue.[42] Further adoption of inter-professional education can help to change attitudes and behaviours and provide a sustainable model to accommodate large numbers of students.[46,47] For example, the non-surgical oncology and palliative care national curricula could be integrated and specialist nurses involved in helping medical students understand different aspects of cancer and end of life care.[11,12,26]

There are several drivers of curriculum development. For example, in 2016, the UK government announced strategies to fund, develop and grow the general practice workforce, to meet the increasing demands of an aging population with complex needs and chronic disease burden.[48] The new UG curriculum for general practice has been developed to complement postgraduate curriculum reflecting how curricula have evolved in relation to health care service and societal needs.[25] Furthermore, the

For peer review only - http://bmjopen.bmj.com/site/about/guidelines.xhtml

introduction of a UK-wide Medical Licencing Assessment in 2022 may influence what medical schools teach and provide opportunities to keep curricular content relevant and inclusive.

Thus, by examining the evidence for need to change or update medical curricula, and through reflection on content and process, Mezirow's concept of transformative learning could be applied by medical educators in curricular development.[49] For instance in Scotland, the Scottish Deans' Medical Curriculum Group (SDMCG) discusses and helps coordinate the development, delivery and evaluation undergraduate curricula. Their aim is to ensure that graduates from each school in Scotland are of an equivalent standard. Their review of the curriculum has also been informed by frameworks on specialist subjects like acute and emergency medicine, dermatology, neurology, palliative care, pharmacology and sexual health.[50]

The curricula developed for foundation and professionalism related subjects recommended their components might be implemented by integration both horizontally and vertically throughout the medical course. These subjects, relevant and applicable to all clinical specialties, should be included in generic outcomes for the specialties and form a framework through the undergraduate years.

It is vital for UG curricula to evolve by keeping abreast with changing clinical needs and advances in diagnostics, investigative and management strategies.

With limited availability of resources and time, the curricula could be split into essential, desirable and nice to know categories to help prioritise areas of teaching and learning. The use of technological advances (e.g. use and sharing of e-learning resources/ webinars) could help provide flexibility and autonomy in how these curricula could be implemented and delivered, if followed.

LIMITATIONS OF THE REVIEW

We conducted a comprehensive search for all relevant articles for this study. However, as with limitations seen in scoping reviews, articles or studies pertinent to the review question may have been unintentionally omitted.[3] The level of evidence for the quality of the developed specialty specific core-curricula could not be graded

For peer review only - http://bmjopen.bmj.com/site/about/guidelines.xhtml

in this search, as done with systematic reviews. However, most curricula were developed with expert consensus and use of a Delphi study, suggesting that efforts were made to establish 'best practice' and core curricular outcomes. The curricular components for individual specialties were variable, making comparisons difficult.

We were nevertheless able to establish common themes and drivers for development of the UG core-curricula for UK medical undergraduates. In addition, the study helped identify gaps in specialty specific curricula like the absence of an overarching RCP curricula, as well as the heterogeneity of the curricular designs between specialties.

CONCLUSIONS

This literature review highlights the status of specialty specific core curricula for medical graduates in UK. This is the first study to provide a comprehensive overview of the curricula developed by specialties and their recommendations on knowledge and skills expected of all UK medical graduates, in alignment with GMC. The review establishes common themes and drivers for development of core-curricula for UK medical undergraduates. The study identifies gaps in development of specific UG curricula as well as the heterogeneity of the curricular designs between specialties and subjects. It would be interesting to see how medical schools are implementing and assessing these curricula and how that is likely to affect future training.

For peer review only - http://bmjopen.bmj.com/site/about/guidelines.xhtml

Contributorship statement:

All 3 authors have made substantial contributions to the planning, conduct, analyses and interpretation of findings, and reporting of the work described in the article, and have agreed to be accountable for all aspects of the work, its accuracy and integrity. MS is responsible for the overall content as guarantor.

MS, RM and GD with RP formulated the review question.

MS screened the titles and abstracts of retrieved citations and MS and RM assessed the full text. MS, RM and GD resolved any ambiguities about inclusion criteria. RM and MS devised the data extraction proforma. MS extracted data and discussed data items with RM and GD. MS contacted study authors for further information.

RM and MS identified common themes, MS conducted the narrative synthesis with RM and GD.

MS wrote the first draft of the manuscript and revised and amended it with RM and GD who also approved the final version to be published. MS is the corresponding author.

The corresponding author attests that all listed authors meet authorship criteria and that no others meeting the criteria have been omitted.

Funding: None

Conflicts of interest: None

Copyright/ Licence for publication:

The Corresponding Author has the right to grant on behalf of all authors and does grant on behalf of all authors, a worldwide licence to the Publishers and its licensees in perpetuity, in all forms, formats and media (whether known now or created in the future), to i) publish, reproduce, distribute, display and store the Contribution, ii) translate the Contribution into other languages, create adaptations, reprints, include within collections and create summaries, extracts and/or, abstracts of the Contribution, iii) create any other derivative work(s) based on the Contribution, iv) to exploit all subsidiary rights in the Contribution, v) the inclusion of electronic links from

For peer review only - http://bmjopen.bmj.com/site/about/guidelines.xhtml

the Contribution to third party material where-ever it may be located; and, vi) licence any third party to do any or all of the above."

Acknowledgements:

Dr Rakesh Patel, Clinical Associate Professor in Medical Education & Honorary Consultant Nephrologist, Faculty of Medicine & Health Sciences, University of Nottingham for guidance on the review question

Lisa Lawrence, Clinical librarian, Burton and Derby Teaching Hospitals NHS Foundation Trust, Derby for conducting the searches for the review

Prof Cathy Bennett, Royal College of Surgeons in Ireland (RCSI), Office of Research and Innovation. Manuscript revision.

Some of the work has been previously presented as oral presentation at:

British Association of Dermatologist (BAD) Teachers of Undergraduate Dermatology Annual Meeting on 6th March 2018

Association for the Study of Medical Education (ASME) Annual Scientific Meeting on 12th July 2018

Ethical approval: None required

Data availability statement: All data relevant to the study are included in the article or uploaded as supplementary information

For peer review only - http://bmjopen.bmj.com/site/about/guidelines.xhtml

References:

1. Promoting excellence: standards for medical education and training. (2015). Manchester, pp.6, 7. Available at: https://www.gmc-uk.org/-/media/documents/Promoting_excellence_standards_for_medical_education_and_training_0715.pdf_61939165.pdf [Accessed 28 Sep. 2018].

2. Medical Licencing Assessment. (2018). General Medical Council. Available at: https://www.gmc-uk.org/education/standards-guidance-and-curricula/projects/medical-licensing-assessment [Accessed 28 Sep. 2018].

3. Outcomes for graduates. (2018).General Medical Council. Available at: https://www.gmc-uk.org/-/media/documents/dc11326-outcomes-for-graduates-2018_pdf-75040796.pdf [Accessed 28 Sep. 2018].

4. Future Hospital: caring for medical patients. (2013). Available at: https://www.rcplondon.ac.uk/projects/outputs/future-hospital-commission [Accessed 28 Sep. 2018].

5. Arksey H, O'Malley L. Scoping studies: towards a methodological framework. Int J Soc Res Methodol. 2005; 8 (1):19-32.

6. The Royal College of Paediatrics. Undergraduate Curriculum for child health. 2015. http://www.rcpch.ac.uk. [Accessed 3 Apr 2017].

7. Royal College of Obstetrics & Gynaecologists. National Undergraduate Curriculum in Obstetrics and Gynaecology.UK.2015. https://www.rcog.org.uk/en/careers-training/specialty-training-curriculum/undergraduate-training-in-og/ [Accessed 3 Apr 2017].

8. Royal College of Psychiatrists. Core Curriculum in Psychiatry. 2011. http://www.rcpsych.ac.uk/workinpsychiatry/faculties/academic/undergraduateteaching/curriculum.aspx. [Accessed 4 Apr 2017].

9. The Royal College of Surgeons. National Undergraduate Curriculum in Surgery.2015.

For peer review only - http://bmjopen.bmj.com/site/about/guidelines.xhtml

https://www.rcseng.ac.uk//media/files/rcs/library-and-publications/non-journal-publications/undergraduate_curriculum_2015-web.pdf. [Accessed 14 May 2017].

10. Baldock C. The Acute Care Undergraduate TEaching (ACUTE) Initiative: consensus development of core competencies in acute care for undergraduates in the United Kingdom. Intensive Care Med. 2005; 31 (12):1627-33.

11. Linklater GT, Bowden J, Pope L et al. Developing learning outcomes for medical students and foundation doctors in palliative care: a national consensus-seeking initiative in Scotland. Med Teach. 2014; 36 (5): 441-446.

12. Paes P, Wee B. A Delphi study to develop the Association for Palliative Medicine consensus syllabus for undergraduate palliative medicine in Great Britain and Ireland. Palliative medicine. 2008; 22 (4): 360-364.

13. Association of British Neurologists. Undergraduate Neurology.2017. http://www.theabn.org/what-we-do/training/undergraduate-neurology.html [Accessed 14 May 2017].

14. British Association of Dermatologists. Undergraduate Dermatology Curriculum.2016 http://www.bad.org.uk/librarymedia/documents/BAD%202016%20revised%20UG%20Curriculum%20(UK).pdf. [Accessed 14 May 2017].

15. Smith CF, Finn GM, Stewart J, McHanwell S. Anatomical Society core regional anatomy syllabus for undergraduate medicine: The Delphi process. J.Anat. 2016; 228 (1):2-14.

16. The Royal College of Pathologists. Pathology Undergraduate Curriculum. 2014. https://www.rcpath.org/resourceLibrary/pathology-undergraduate-curriculum-.html. [Accessed 3 Apr 2017].

17. Jackson, A.A., Human nutrition in medical practice: the training of doctors. Proc Nut Soc. 2001; 60 (2): 257-63.

For peer review only - http://bmjopen.bmj.com/site/about/guidelines.xhtml

18. Stirrat GM, Johnston C, Gillon R. Boyd K. Medical ethics and law for doctors of tomorrow: the 1998 Consensus Statement updated. J med ethics. 2010; 36 (1):55-60.

19. Masud T, Blundell A, Gordon AL, et al. European undergraduate curriculum in geriatric medicine developed using an international modified Delphi technique. Age and ageing. 2014; 43 (5):695-702.

20. Bachmann C, Abramovitch H, Barbu CG, et al. A European consensus on learning objectives for a core communication curriculum in health care professions. Patient Educ Couns. 2013; 93 (1):18-26.

21. Noble LM, Scott-Smith W, O'Neill B, Salisbury H. Consensus statement on an updated core communication curriculum for UK undergraduate medical education. 2018; 101 (9) 1712-19.

22. British Geriatrics Society Recommended Curriculum in Geriatric Medicine for MedicalUndergraduates.2013. http://www.bgs.org.uk/generalinformation171/secttrainees/generalinfo/undergraduatecurriculum. [Accessed 14 May 2017].

23. Woolf AD, Walsh NE. Global core recommendations for a musculoskeletal undergraduate curriculum. Ann Rheum Dis. 2004; 63:517-24.

24. Coady D, Walker D, Kay L. Regional Examination of the Musculoskeletal System (REMS): a core set of clinical skills for medical students. Rheumatology. 2004; 43: 633–639.

25. Royal College of General Practitioners. (2018).Teaching general practice. Guiding principles for undergraduate general practice curricula in UK medical schools. [Accessed 3 March 2019].

26. The Royal College of Radiologists. Medical undergraduate non-surgical oncologycurriculum.2014. https://www.rcr.ac.uk/clinical-oncology/undergradute-oncology. [Accessed 4 May 2017].

27. The Royal College of Radiologists. Undergraduate radiology curriculum second edition. London, 2017. www.rcr.ac.uk. [Accessed 4 Apr 2017].

For peer review only

For peer review only - http://bmjopen.bmj.com/site/about/guidelines.xhtml

28. Royal College of Ophthalmologists. "Eyes & Vision Curriculum" for Undergraduate and Foundation Doctors. 2015. https://www.rcophth.ac.uk/wp-content/uploads/2014/07/Undergraduate-and-Foundation-doctors-curriculum.pdf.[Accessed 3 Apr 2017].

29. Public Health Educators in Medical Schools. Undergraduate Public Health Curriculum for UK Medical Schools. Consensus Statement 2014. http://www.fph.org.uk/uploads/PHEMS booklet web.pdf. [Accessed 14 May 2017].

30. Ross S, Maxwell S. Prescribing and the core curriculum for tomorrow's doctors: BPS curriculum in clinical pharmacology and prescribing for medical students. Br. J. Clin. Pharmacol. 2012; 74 (4):644-61.

31. Core curriculum for sociology in undergraduate medical education. Behavioural & Social Sciences Teaching in Medicine (BeSST) Sociology SteeringGroup.2016.http://www.ed.ac.uk/usher/news-events/news-2016/core-curriculum-sociology-medical-education. [Accessed 20 Apr 2017].

32. Turner K, Brewster S. Undergraduate urology: A survey of current provisions and guidelines for a core curriculum. BJU Int. 2002; 90 (4): 475.

33. The British Association of Urological Surgeons. An undergraduate syllabus for urology.2013.https://www.baus.org.uk/_userfiles/pages/files/professionals/education/Undergraduate%20Syllabus.pdf. [Accessed 14 May 2017].

34. Lyoyd S, Tan ZE, Taube M-A, Doshi J. Development of an ENT undergraduate curriculum using a Delphi survey. Clin Otolaryngol. 2014; 39:281-288.

35. Bleakley A, Marshall R, Brömer R. Toward an aesthetic medicine: developing a core medical humanities undergraduate curriculum. J med humanit. 2006; 27 (4):197-213.

36. WHO Multi-professional Patient Safety Curriculum Guide.2011. World Health Organization.http://www.who.int/patientsafety/education/mp_curriculum_guide/en/ [Accessed 3 Apr 2017].

For peer review only - http://bmjopen.bmj.com/site/about/guidelines.xhtml

37. Burge SM. Undergraduate medical curricula: are students being trained to meet future service needs? Clin Med.2003; 3 (3): 243-6.

38. Association of British Neurologists Training & Education Subcommittee. Clinical Neurology Medical Undergraduate Curriculum UK Medical School Survey.2002. http://www.theabn.org/media/Med%20Sch%20Neuro%20Survey%202002-3.pdf [Accessed 30/7/14] and CM Wiles, P Bain (Personal communications 2011).

39. Wilkinson IMS. A survey of undergraduate teaching of clinical neurology in the UK 1990. J Neurol Neurosurg Psych. 1991; 54:266-68.

40. General Medical Council (2010) Standards for curricula and assessment systems. 2010. http://www.gmc-uk.org/education/postgraduate/standards_forcurricula_and_assessment_systems.asp. [Accessed 14 May 2017].

41. McIntosh N, Olver RE, Haddad, D. What is core? Guidelines for the core curriculum in paediatrics. Med Educ.1997; 31(5): 354-8.

42. Hirsh D, Gaufberg E, Ogur B, Cohen P, Krupat E, Cox M, Pelletier S, Bor D. Educational outcomes of the Harvard Medical School-Cambridge integrated clerkship: a way forward for medical education. Acad Med. 2012. 87(5): 643-50.

43. Voss JD, May NB, Schorling JB et al. Changing conversations: teaching safety and quality in residency training. Academic Medicine.2008; 83:1080-7.

44. Copeland C, Fisher J, Teodorczuk A. Development of an international undergraduate curriculum for delirium using a modified delphi process. Age and Ageing. 2018; 47. 131-73.

45. Wakefield RJ, Weerasingheb A Tung P, et al. The development of a pragmatic, clinically driven ultrasound curriculum in a UK medical school Medical Teach 2018; 40(6): 600–606.

For peer review only

46. Ponzer S, Hylin U, Kusuoffsky A et al. Interprofessional training in the context of clinical practice: goals and students' perceptions on clinical education wards. Medical Education. 2004; 38(7):727-36.

47. Anderson ES, Lennox A. The Leicester Model of Interprofessional education: developing, delivering and learning from student voices for 10 years. J Interprof Care.2009; 23(6):557-73.

48. NHS England. General Practice Forward View.2016. https://www.england.nhs.uk/gp/gpfv/ [Accessed 22 May 2017].

49. Mezirow J and associates (eds). Fostering Critical Reflection in Adulthood. 1990. Jossey-Bass, San Francisco, CA.

50. Scottish Deans' Medical Curriculum Group. The Scottish Doctor: Learning Outcomes for the medical undergraduate in Scotland: a Foundation for Competent and Reflective Practitioners. 2008: Dundee. UK. http://www.scottishdoctor.org/resources/scotdoc3.pdf[Accessed 22 May 2017]

For peer review only - http://bmjopen.bmj.com/site/about/guidelines.xhtml

Table 1: Electronic data collection table of specialty or subject with curriculum in alphabetical order

	Specialty with curriculum	Year	Organization/Body	Alignment with GMC outcomes
**1.**	**Acute care**[10]	2005	Resuscitation Council UK	Y
**2.**	Regional **Anatomy**[15]	2016	Anatomical Society	Y
**3.**	**Clinical pharmacology and prescribing**[30]	2012	British Pharmaceutical Society	Y
**4. a**	**Core communications curriculum**[20]	2013	European study using Delphi process	
**b**	**Communications curricula**[21]	2018	U. K. Council for Clinical Communication Skills Teaching in Undergraduate Medical Education	Y
**5.**	**Dermatology**[14]	2016	British association of dermatologists	Y
**6.**	**ENT**[34]	2014	A Delphi survey (UK) of ENT consultants and specialist registrars, accident and emergency consultants and	Y

For peer review only - http://bmjopen.bmj.com/site/about/guidelines.xhtml

			specialist registrars, general practitioners and paediatricians.	
**7.**	**General Practice**[25]	2018	Royal college of general practitioners	Y
**8. a**	European undergraduate curriculum in **geriatric medicine**[19]	2014	European Union of Medical Specialists	Y
**b**	**Geriatric medicine** for undergraduates[22]	2013	British Geriatrics Society	Y
**9.**	**Medical Ethics and Law** [18]	2010	Medical Education Working Group of the Institute of Medical Ethics and associated signatories	Y
**10.**	**Medical Humanities**[35]	2006	Peninsula medical school	Unclear
**11.a**	**Musculoskeletal system (MSK)**[23]	2004	The Bone and Joint Decade ((BJD) Undergraduate Curriculum Development Group	Y
**b**	**Regional Examination of the Musculoskeletal System (REMS)**[24]	2004	UK representation of the four specialties- Rheumatologists,	Y

For peer review only - http://bmjopen.bmj.com/site/about/guidelines.xhtml

			Orthopaedic Surgeons, Geriatricians and General Practitioners	
**12.**	**Neurology**[13]	2017	Association of British Neurologists	Y
**13.**	Human **Nutrition**[17]	2001	National Nutrition Task Force	Y
**14..**	**Obstetrics & Gynaecology**[7]	2015	Royal College of Obstetricians and Gynaecologists (RCOG)	Y
**15.**	**Ophthalmology**[28]	2015	Royal college of Ophthalmologists	Y
**16.**	**Oncology**[26]	2014	The Royal College of Radiologists	Y
**17.**	**Paediatrics**[6]	2015	Royal college of paediatrics and child health	Y
**18.a**	**Palliative Care**[11]	2014	Scottish Palliative Medicine Curriculum Development Group	Y
**b**	**Palliative Medicine**[12]	2008	Association for Palliative Medicine	Y
**19.**	**Pathology**[16]	2014	The Royal College of Pathologists	Y
**20..**	**Patient Safety**[36]	2011	World Health Organization	Y

For peer review only

For peer review only - http://bmjopen.bmj.com/site/about/guidelines.xhtml

**21.**	**Psychiatry**[8]	2011	Royal college of psychiatrists	Y
**22.**	**Public Health**[29]	2014	Faculty of Public Health	Y
**23.**	**Radiology**[27]	2017	Royal college of radiologists	Y
**24.**	**Sociology**[31]	2016	Behavioural & Social Sciences Teaching in Medicine (BeSST) Sociology Steering Group	Y
**25.**	**Surgery**[9]	2015	Royal college of Surgeons	Y
**26.a**	**Urology**[32]	2002	Journal article in Urology	Unclear
**b**	**Urology syllabus**[33]	2012	British Association of Urological Surgeons	Unclear

For peer review only - http://bmjopen.bmj.com/site/about/guidelines.xhtml

Supplementary material

Figure's legend

Figure 1: PRISMA flow diagram of the scoping review

Appendix 1: Example of search strategy (MEDLINE)

For peer review only

For peer review only - http://bmjopen.bmj.com/site/about/guidelines.xhtml

Figure 1: PRISMA (preferred reporting items for systematic reviews and meta-analysis) flow diagram of the scoping review

UG= undergraduate

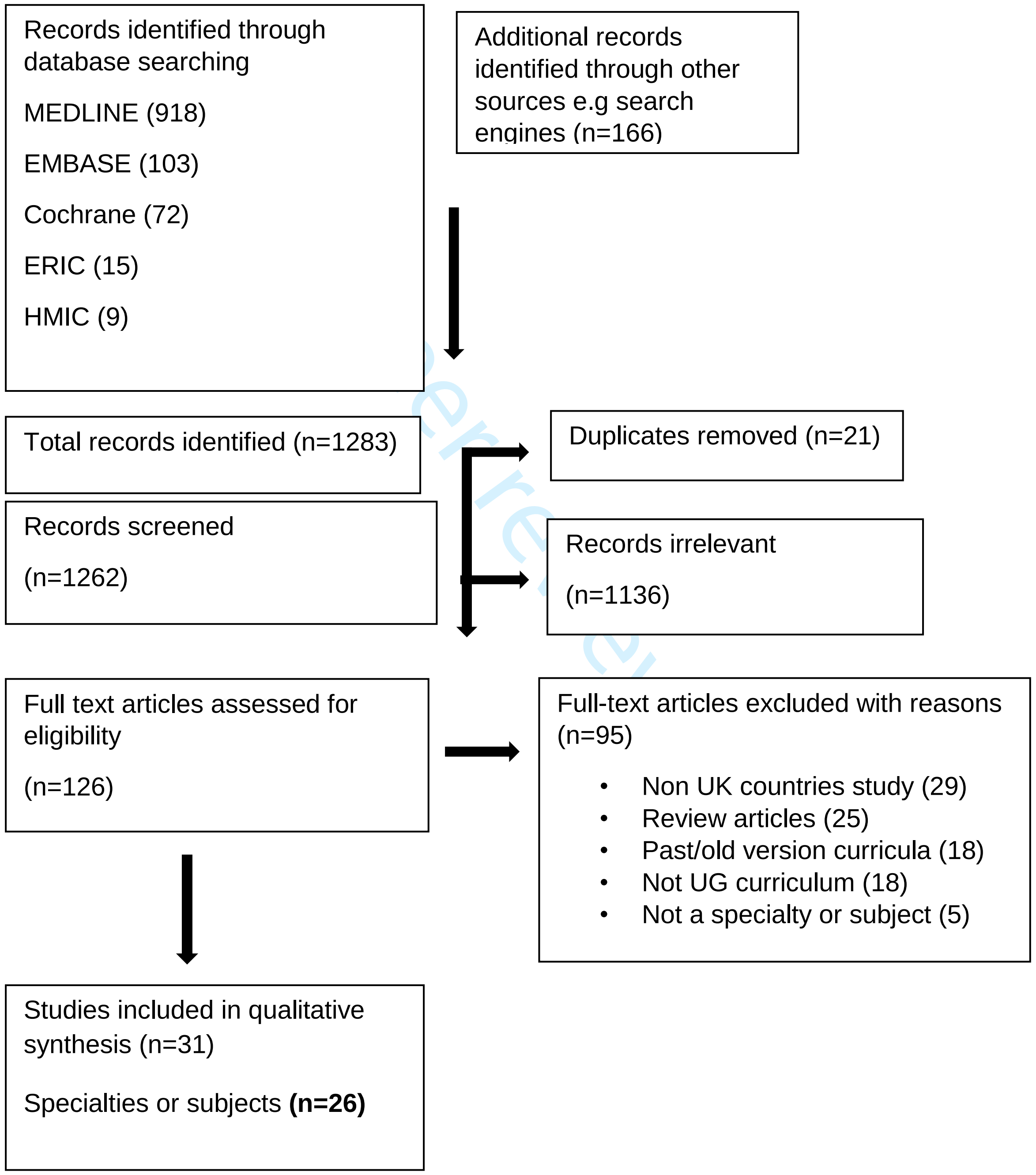

Example Search Strategy for Appendix

NICE Healthcare Databases Advanced Search MEDLINE 1946 to present (Provided by Proquest).

Strategy 606888

#	Database	Search term	Results
1	Medline	"EDUCATION, MEDICAL, UNDERGRADUATE"/	22398
2	Medline	("medical education").ti,ab	36006
3	Medline	(undergraduate).ti,ab	29224
4	Medline	("medical student").ti,ab	6528
5	Medline	"STUDENTS, MEDICAL"/	30311
6	Medline	(2 AND 3)	3401
7	Medline	(1 OR 4 OR 5 OR 6)	48667
8	Medline	CURRICULUM/	70249
9	Medline	(curricul*).ti,ab	48931
10	Medline	(8 OR 9)	93842
11	Medline	(core OR influenc* OR design* OR develop* OR deliver* OR enabl* OR barrier* OR process* OR factor*).ti,ab	9645339
12	Medline	"REFERENCE STANDARDS"/	40365
13	Medline	(standard*).ti,ab	1110534
14	Medline	(12 OR 13)	1133896
15	Medline	(11 OR 14)	10194258
16	Medline	DERMATOLOGY/	17224

For peer review only

17	Medline	(dermatology).ti,ab	23551
18	Medline	(16 OR 17)	33537
19	Medline	exp "UNITED KINGDOM"/	350174
20	Medline	(UK OR "United Kingdom" OR "Great Britain" OR GB OR England OR Wales OR "Northern Ireland" OR Scotland).ti,ab	205051
21	Medline	(19 OR 20)	459845
22	Medline	(7 AND 10 AND 15)	8130
23	Medline	22 [DT 1994-2019]	7323
24	Medline	(7 AND 10 AND 15 AND 18)	57
25	Medline	24 [DT 1994-2019]	52
26	Medline	(7 AND 10 AND 15 AND 21)	788
27	Medline	26 [DT 1994-2019]	718
28	Medline	(7 AND 10 AND 15 AND 18 AND 21)	8
29	Medline	28 [DT 1994-2019]	8

For peer review only

Preferred Reporting Items for Systematic reviews and Meta-Analyses extension for Scoping Reviews (PRISMA-ScR) Checklist

SECTION	ITEM	PRISMA-ScR CHECKLIST ITEM	REPORTED ON PAGE #
**TITLE**			
Title	1	Identify the report as a scoping review.	1
**ABSTRACT**			
Structured summary	2	Provide a structured summary that includes (as applicable): background, objectives, eligibility criteria, sources of evidence, charting methods, results, and conclusions that relate to the review questions and objectives.	2
**INTRODUCTION**			
Rationale	3	Describe the rationale for the review in the context of what is already known. Explain why the review questions/objectives lend themselves to a scoping review approach.	4
Objectives	4	Provide an explicit statement of the questions and objectives being addressed with reference to their key elements (e.g., population or participants, concepts, and context) or other relevant key elements used to conceptualize the review questions and/or objectives.	4
**METHODS**			
Protocol and registration	5	Indicate whether a review protocol exists; state if and where it can be accessed (e.g., a Web address); and if available, provide registration information, including the registration number.	Not applicable
Eligibility criteria	6	Specify characteristics of the sources of evidence used as eligibility criteria (e.g., years considered, language, and publication status), and provide a rationale.	5 and 6
Information sources*	7	Describe all information sources in the search (e.g., databases with dates of coverage and contact with authors to identify additional sources), as well as the date the most recent search was executed.	5 and appendix 1
Search	8	Present the full electronic search strategy for at least 1 database, including any limits used, such that it could be repeated.	5 and appendix 1
Selection of sources of evidence†	9	State the process for selecting sources of evidence (i.e., screening and eligibility) included in the scoping review.	5,6
Data charting process‡	10	Describe the methods of charting data from the included sources of evidence (e.g., calibrated forms or forms that have been tested by the team before their use, and whether data charting was done independently or in duplicate) and any processes for obtaining and confirming data from investigators.	6 and Data from individual studies extracted into Table 1
Data items	11	List and define all variables for which data were sought and any assumptions and simplifications made.	6 and Table 1

St. Michael's
Inspired Care.
Inspiring Science.

For peer review only - http://bmjopen.bmj.com/site/about/guidelines.xhtml

SECTION	ITEM	PRISMA-ScR CHECKLIST ITEM	REPORTED ON PAGE #
Critical appraisal of individual sources of evidence§	12	If done, provide a rationale for conducting a critical appraisal of included sources of evidence; describe the methods used and how this information was used in any data synthesis (if appropriate).	Study quality appraisal not applicable.
Synthesis of results	13	Describe the methods of handling and summarizing the data that were charted.	Table 1
**RESULTS**			
Selection of sources of evidence	14	Give numbers of sources of evidence screened, assessed for eligibility, and included in the review, with reasons for exclusions at each stage, ideally using a flow diagram.	5 and PRISMA diagram
Characteristics of sources of evidence	15	For each source of evidence, present characteristics for which data were charted and provide the citations.	Table 1 and page 7
Critical appraisal within sources of evidence	16	If done, present data on critical appraisal of included sources of evidence (see item 12).	Not applicable
Results of individual sources of evidence	17	For each included source of evidence, present the relevant data that were charted that relate to the review questions and objectives.	7-10 (results section)
Synthesis of results	18	Summarize and/or present the charting results as they relate to the review questions and objectives.	7-10 Narrative synthesis of results
**DISCUSSION**			
Summary of evidence	19	Summarize the main results (including an overview of concepts, themes, and types of evidence available), link to the review questions and objectives, and consider the relevance to key groups.	10-13
Limitations	20	Discuss the limitations of the scoping review process.	13
Conclusions	21	Provide a general interpretation of the results with respect to the review questions and objectives, as well as potential implications and/or next steps.	13
**FUNDING**			
Funding	22	Describe sources of funding for the included sources of evidence, as well as sources of funding for the scoping review. Describe the role of the funders of the scoping review.	14

JBI = Joanna Briggs Institute; PRISMA-ScR = Preferred Reporting Items for Systematic reviews and Meta-Analyses extension for Scoping Reviews.
* Where *sources of evidence* (see second footnote) are compiled from, such as bibliographic databases, social media platforms, and Web sites.
† A more inclusive/heterogeneous term used to account for the different types of evidence or data sources (e.g., quantitative and/or qualitative research, expert opinion, and policy documents) that may be eligible in a scoping review as opposed to only studies. This is not to be confused with *information sources* (see first footnote).
‡ The frameworks by Arksey and O'Malley (6) and Levac and colleagues (7) and the JBI guidance (4, 5) refer to the process of data extraction in a scoping review as data charting.
§ The process of systematically examining research evidence to assess its validity, results, and relevance before using it to inform a decision. This term is used for items 12 and 19 instead of "risk of bias" (which is more applicable to systematic reviews of interventions) to include and acknowledge the various sources of evidence that may be used in a scoping review (e.g., quantitative and/or qualitative research, expert opinion, and policy document).

For peer review only - http://bmjopen.bmj.com/site/about/guidelines.xhtml

BMJ Open

DO WE NEED A CORE-CURRICULUM FOR MEDICAL STUDENTS? A SCOPING REVIEW

Journal:	*BMJ Open*
Manuscript ID	bmjopen-2018-027369.R3
Article Type:	Research
Date Submitted by the Author:	09-Jul-2019

Complete List of Authors:	Sharma, Maulina; University of Nottingham, Medical Education Centre, School of Medicine; University Hospitals of Derby & Burton NHS Foundation Trust , Dermatology<br>Murphy, Ruth; University of Nottingham, School of Medicine<br>Doody, Gillian; University of Nottingham, Medical Education Centre, School of Medicine

**Primary Subject Heading**:	Medical education and training
Secondary Subject Heading:	Medical education and training
Keywords:	Undergraduate, MEDICAL EDUCATION & TRAINING, United Kingdom, Core, Curriculum

SCHOLARONE™
Manuscripts

DO WE NEED A CORE-CURRICULUM FOR MEDICAL STUDENTS? A SCOPING REVIEW

Maulina Sharma, Ruth Murphy, Gillian A Doody

Medical Education Unit, University of Nottingham, School of Medicine, Nottingham, NG7 2UH, UK

Maulina Sharma, Consultant Dermatologist, Honorary Clinical Associate Professor, University of Nottingham, School of Medicine, Department of Dermatology London Road Community Hospital, University Hospitals of Derby & Burton NHS Foundation Trust, Derby DE1 2QY, UK

Ruth Murphy, Consultant Dermatologist, University of Nottingham, School of Medicine, Nottingham, NG7 2UH, UK

Gillian A Doody, Dean of Medical Education, Medical Education Unit, University of Nottingham, School of Medicine, Nottingham, NG7 2UH, UK

Corresponding author:

M Sharma email : msxms16@nottingham.ac.uk

Tel no: 01332 254653

Word count: 3006

Keywords: core curriculum, medical undergraduates, UK

For peer review only - http://bmjopen.bmj.com/site/about/guidelines.xhtml

ABSTRACT

Objective

The General Medical Council (GMC) recommends medical schools to develop and implement curricula enabling students to achieve the required learning outcomes. UK medical schools follow the GMC's Outcomes for Graduates, which are generic. GMC plans to introduce a national Medical Licencing Assessment (MLA) for medical graduates wanting to practice medicine in the UK in 2022. With no standardised or unified undergraduate (UG) curriculum in UK, various specialities have expressed concerns about not being represented in medical schools and developed specialty-specific core-curricula.

The aim of this review was to identify learned bodies who have developed a core-curriculum for UK medical schools and highlight the drivers, gaps and future approaches to curricular development and implementation.

Methods

A literature search was conducted using online databases (EMBASE, MEDLINE, ERIC, HMIC, PubMed and CDSR), search engines, and related websites (Google & Google Scholar, Department of Health, GMC and BMA) for relevant articles from 1996 to 5th March 2019 (~20 years). A methodological framework to map the key concepts of undergraduate medical curriculum was followed. Any relevant body with a core-curriculum for UK medical undergraduates was included.

Results

A total of 1283 articles were analysed with 31 articles included in the qualitative synthesis, comprising of 26 specialties (clinical n=18, foundation subjects n=4, professionalism related n=4). WHO, European and national (e.g. Royal Colleges of UK) specialty bodies provided specific core learning outcomes for medical graduates. Patient safety, disease burden, needs of society and inadequate preparedness of medical graduates were drivers for development of these curricula.

Conclusions

This is the first comprehensive review of literature on undergraduate core-curricula recommending minimum standards on knowledge and skills, in alignment with

For peer review only - http://bmjopen.bmj.com/site/about/guidelines.xhtml

GMC's outcomes for graduates for all UK medical students. Adopting and assessing unified standards would help reduce variability across UK medical schools for both generic and specialty-specific competencies.

Strengths and limitations of the study

- Scoping review of literature as the methodological approach in the study helped identify, categorise and provide a narrative synthesis of the key aspects on core-curricula for UK medical students.
- A strength of the study is that it is the first comprehensive review on core-curricula for UK medical students which could help medical schools in curriculum mapping and implementation.
- The scoping study highlighted gaps in specific undergraduate curricula as well as the heterogeneity of the curricular designs between specialties and subjects.
- A limitation of the study is that heterogeneity in the curricular designs for specialties made comparisons between them difficult.
- As with limitations seen in other scoping reviews, articles or studies pertinent to the review question may have been unintentionally omitted.

.

For peer review only

For peer review only - http://bmjopen.bmj.com/site/about/guidelines.xhtml

INTRODUCTION

The General Medical Council (GMC) set out the Promoting Excellence document in 2016, which outlines a set of standards for all stages of medical education and training, including undergraduate (UG) medical education.[1] These standards should ensure that medical schools develop and implement curricula to enable students to achieve learning outcomes required as graduates and practise safely and competently. The GMC also plans to introduce a Medical Licencing Assessment (MLA) in 2022 applicable to medical graduates who want to practice medicine in the UK.[2] Specialities have expressed concern about medical curricula as medical schools in the UK currently organise their own curricula and assessments based on GMC's Outcomes for Graduates, which can be viewed as generic.[3] This may result in scanty representation of specialist knowledge and skills within the curriculum. Once the students successfully graduate, the GMC grants them a licence to practice.

In the UK there is a growing need for development of general practitioners, who treat all common medical conditions and refer patients into secondary care for urgent or specialist treatment. Also, a 'Future Hospital Commission' report (FHC), through the Royal College of Physicians (RCP) in 2013 promoted the re-introduction of generalism into medical training.[4] This means that medical education and training of doctors in UK needs to address the current and future demographic of patients.

With the generalist trend, as well as plans to introduce the MLA for all medical graduates in the UK, medical schools may be influenced on what they teach and assess. There is currently no unified or standardised UG medical curriculum in the UK. Various specialities have expressed concerns about not being adequately represented in medical school curricula and have developed specialty-specific core-curricula.

Our objective was to perform a scoping review of literature to identify the learned bodies who have developed a core-curriculum for UK medical schools and to use this information to highlight the drivers, gaps and future approaches to curricular development and implementation.

For peer review only - http://bmjopen.bmj.com/site/about/guidelines.xhtml

METHODS

We performed a scoping review using the methodological framework as outlined by Arksey and O'Malley.[5] The framework was used to: identify the specialties, or subjects which recommended and had developed an undergraduate core-curriculum for UK medical schools; select and search for relevant studies; chart and collate the data, and summarise the results.

Search strategy

A literature search was conducted using online databases (EMBASE, MEDLINE, HMIC (searched via NICE Healthcare Databases Advanced Search), PubMed, The Cochrane Library and ERIC (Education Resources Information Centre). Other online search engines (Google & Google Scholar) and websites of Department of Health, GMC and BMA were also searched for relevant articles from 1996 to May 2017 (i.e. the past 20 years). The literature search was updated up to 5th March 2019 for any further recent relevant articles.

Keywords or terms used in the searched included: General Medical Council (GMC), medical student, medical education, undergraduate, curriculum, standards and national (UK and individual countries e.g. England). A draft of the full search strategy is included in Appendix 1.

Electronic search results were managed using a reference manager. Titles and abstracts of all citations were first screened by the author (MS) and those that were not related to undergraduate curricula or the UK were excluded. Full articles were requested from the library, when it was not possible to eliminate them from reading either their title or abstract. The full text of all potentially eligible articles were then obtained and assessed against the inclusion criteria (MS and RM). Where the full text of an article was not available the authors were contacted to request a copy. Any ambiguities about whether a study met the inclusion criteria were resolved by a consensus between authors (MS, RM, and GD).

Eligibility criteria

For peer review only - http://bmjopen.bmj.com/site/about/guidelines.xhtml

Inclusion criteria were all published studies, reports or articles which had a recommended core-curriculum for a specialty or subject for UK medical undergraduates on a national level. Any international or national body, society, college or organisation specific and pertinent to UK medical undergraduate education was included. For curricula which had been revised or updated, the most recent (up to 05/03/19) were included.

Exclusion criteria were articles pertaining to core-curricula for postgraduate courses and those not specific to UK medical undergraduates. Due to the study being relevant to UK setting, non-English language literature was excluded.

Patient and Public involvement: As this was a scoping review of available undergraduate core-curricula, patients or public were not involved.

Data management

Data were collated and reported using PRISMA flow diagram (Figure 1). Characteristics of each study were recorded in an electronic data collection table and included the name of specialty or subject, in alphabetical order, organisation or body developing the curriculum, year of the curriculum update, and whether the studies were aligned to GMC's learning outcomes (Table 1).

The key concepts derived were broadly grouped into the following categories: grouping of core-curricula and their timing within the school course; organisations involved in development of the core-curricula; drivers for development of core-curricula; and curriculum overlaps and gaps.

RESULTS

The initial search identified a total of 1283 articles, after duplicates were removed (n=21). All articles were screened and 126 were initially highlighted as of interest. Of these, 95 were subsequently excluded, with reasons recorded. A total of 31 articles, published since 2002, and describing core-curricula recommendations for UK medical undergraduates were then included in the narrative synthesis. These comprised a total of 26 specialties or subjects (Figure 1). Of these 26 specialties, 5 specialties had additional relevant articles (Table1).

For peer review only - http://bmjopen.bmj.com/site/about/guidelines.xhtml

Narrative synthesis of results

Having identified publications from learned bodies that had developed a core-curriculum for UK medical schools, we identified the following themes and drivers for curricular development and implementation, and summarised areas where there were gaps and overlaps which may influence future approaches to curricular development and implementation.

i) Grouping of curricula and timing within the school course

The curricula developed and recommended for UK medical schools could be grouped into 3 main groups: clinical specialty based (n=18) (e.g. surgery, dermatology); foundation subjects (n=4) (e.g. anatomy, pharmacology) and subjects related to professionalism (n=4) (e.g. communication skills) (Table 1).

Clinical specialty curricula were applicable during clinical years of training, e.g. paediatrics, obstetrics & gynaecology, psychiatry, surgery, acute medical care, resuscitation and end of life care.[6-12] Some specialties had developed a curriculum with a system-based approach e.g. neurology and dermatology.[13,14] Foundation subjects like anatomy, pathology and nutrition as well as curricula related to professionalism, such as medical ethics and law, were developed so as to be relevant throughout all medical school training.[15-18]

ii) Organisations involved in development of individual core-curricula

Curricula were developed by international, national and regional organisations with participation from relevant stakeholders. Consensus agreement derived from a Delphi process was the preferred method of achieving core-curricular recommendations.[10, 11, 15, 18,-21,]

Four curricula, namely communications skills, geriatrics, musculoskeletal system and palliative care had their core-curriculum co-developed by two different international and national bodies. The international recommendations encompassed the national guidance.[19-24]

The Royal Colleges of the UK developed specialty specific core-curricula for 9 specialties (e.g. general practice, pathology, radiology, surgery) [6-9, 16, 25-28]

For peer review only - http://bmjopen.bmj.com/site/about/guidelines.xhtml

National specialist consultant societies and bodies developed 14 specialty specific curricula (e.g. dermatology, medical ethics, palliative care) [10—15, 17, 18, 21-24, 29, 30, 31].

National expert consensus through a survey of consultants was used to develop guidelines for core curricula in urology and ENT, whilst a separate urology syllabus for medical undergraduates was developed by the British Association of Urological Surgeons.[32-34] At a regional level, one medical school developed and recommended a curriculum for medical humanities.[35]

iii) Drivers for development of core-curricula

a) Patient safety

Patient safety was a common theme in the development of core-curricula for clinical specialties, foundation subjects, in addition to patient expectations of a graduate in terms of medical ethics and law.[15,18,21,27,22,23] The World Health Organisation (WHO), have published the multi-professional patient safety curriculum guide to aid teaching of patient safety in universities and schools, including medicine.[36] The ACUTE initiative identified the core set of competencies for resuscitation and care of acutely ill patients that all undergraduate trainees require to be competent in at the point of graduation.[10] This was designed to address the sub-optimal care of the critically ill in hospital where contributory factors included recognition of critical illness, lack of knowledge, a lack of supervision, failure to seek advice and poor communication.[10] Similarly, the British Pharmacology Society (BPS) prepared a curriculum to standardise the learning objectives and recommended introduction of prescribing safety assessments in all medical schools, in alignment with GMC's outcomes.[30]

b) Disease burden and needs of society

In the UK, cancer affects 1 in 3 people.[26] The Royal College of Radiologists, who developed the UG radiology curriculum, also developed the non-surgical oncology curriculum. With collaboration from the Royal College of Physicians' Joint Collegiate Council for Oncology (JCCO), the UG curriculum with its joint approach aims to address competencies in a multidisciplinary setting towards patients affected with cancer and their needs.[26, 27]

For peer review only - http://bmjopen.bmj.com/site/about/guidelines.xhtml

Societal requirements of an ageing, frail population, with complex problems related to independence and mobility were important factors for the development of a core-curricula for geriatric medicine and the musculoskeletal system. [22-24]

The National Health Service (NHS) of the UK is underpinned by General Practitioners (GP), who remain the first point of contact for patients and provide long-term continuity of care. To provide a sustainable service to patients, UK needs approximately half of medical school graduates to pursue a career in family medicine or general practice.[25] In 2018, the Royal College of General Practitioners (RCGP) with the Society for Academic Primary Care (SAPC) collaborated to develop the GP curriculum as guidance to UK medical schools on the design and delivery of teaching of general practice to help bridge the gap between societal demands and needs. [25]

Public healthcare prevention and promotion, human nutrition and its relevance to health and disease also had specific UG curricula.[17, 25]

c) Lack of representation and inadequate preparedness amongst graduates

Multiple specialties, through national surveys across UK medical schools, felt a lack of adequate representation, teaching or assessments in medical school curricula.[13,14,32-34,37] These concerns were echoed by feelings of inadequacy amongst junior doctors and general practitioners due to a lack of undergraduate training during in some specialties, and some of these specialties subsequently developed their own curricula.[13,38,39] This is particularly seen in specific core-curricula like ENT and Dermatology, where related diseases and conditions are common presentations in UK primary care.[14,34] This has led such specialties to develop specialty specific learning objectives, defining minimum knowledge and skills to be attained.

iv) Curriculum: overlaps and gaps

The General Medical Council, based on a review of curriculum theories, its relevance and context with regards to medical education and its stakeholders i.e. students,

For peer review only - http://bmjopen.bmj.com/site/about/guidelines.xhtml

teachers and regulators, defines “a curriculum as a statement of the intended outcomes, encompassing content, teaching, learning and assessment methods, feedback and supervision as part of the educational programme”.[40]

This scoping review found that the Royal Colleges of the UK, such as the Royal College of Surgeons (RCS) and Obstetrics and Gynaecology (RCOG) covered the curricular requirements comprehensively by highlighting content and how to achieve the learning objectives with structure and assessment methods.[7, 9]

However, many clinical and foundation subjects suggested medical schools determine how best to incorporate content within their medical course to allow flexibility in their implementation, delivery and assessment. Hence, they were focussed primarily on syllabus content.[13,14,28,34]

Despite representation of surgical sub-specialties like ENT and urology in the RCS curriculum, both sub-specialities developed specific outcomes for medical school curricula.[32-4] Overlapping of core-content was also seen between more established curricula like geriatrics[2] and psychiatry.[8] Interestingly, there was no specific UG curriculum for medicine even though the Royal College of Physicians (RCP), sets out postgraduate standards for specialty physicians in UK.

DISCUSSION

We used a structured literature search to identify publications which described core-curriculum recommendations for a specialty or subject for UK medical undergraduates. We identified common factors and themes, and bodies who have developed a core-curriculum for UK medical schools, grouping specialties into clinical, foundation and professionalism related subjects.

Medical schools have an enormous task of continuously responding to changes in clinical practice, keeping the curriculum in context and ensuring that students are not overburdened by content. From our review of the available literature, we demonstrated that most of the available specialty specific core-curricula had been developed through international and national expert consensus, advising on minimum standards on core knowledge and skills to be achieved by medical

For peer review only - http://bmjopen.bmj.com/site/about/guidelines.xhtml

graduates and aligned themselves to the Outcomes for Graduates document. The 'core-knowledge' in specialty specific curricula could help develop minimum standards and provide quality assurance for both generic and specialty-specific areas across all UK medical schools. The drivers to curriculum development included patient safety, disease burden and needs of society, and lack of representation or inadequate preparedness amongst graduates. We also identified areas where there were gaps or overlaps in some specialities' curricula. Though the GMC recommends a 'core curriculum' for UG medical education, medical educators have found it challenging to define what is meant by core, what needs to be included and its relevance to the bigger context of competence for graduating medical students.[1,2,3]

Whilst medical schools align their curricula to the generic 'Outcomes for Graduates', specialty representation and mapping of burden of disease in curricula is challenging, and how individual providers of medical education map curriculum content, learning outcomes and assessments for specialties is not explicit.[41,42] As Voss et al have noted, 'Improving patient safety and quality in health care is one of medicine's most pressing challenges'.[43] The GMC has also highlighted these areas in the new Outcomes for Graduates.[3]

In the review, it was observed that there was duplication and overlapping of some specialties and subjects with their core-curricular recommendations. For this review, curricula developed on topics within specialties were excluded. For example, a curriculum for delirium has been developed which is a topic covered within psychiatry and geriatrics specialty.[44]Similarly, recently, an ultrasound curriculum[45] for medical students has been developed in addition to the national UG curriculum for radiology. Perhaps necessarily, certain clinical conditions demand a multi-disciplinary approach. An integrated approach to combine specialties would help reduce content overload, whilst keeping teaching and learning contextual. The introduction of longitudinal integrated clerkship in medical schools is looking to address this issue.[42] Further adoption of inter-professional education can help to change attitudes and behaviours and provide a sustainable model to accommodate large numbers of students.[46,47] For example, the non-surgical oncology and palliative

For peer review only - http://bmjopen.bmj.com/site/about/guidelines.xhtml

care national curricula could be integrated and specialist nurses involved in helping medical students understand different aspects of cancer and end of life care.[11,12,26]

There were several drivers of curriculum development. For example, in 2016, the UK government announced strategies to fund, develop and grow the general practice workforce, to meet the increasing demands of an aging population with complex needs and chronic disease burden.[48] The new UG curriculum for general practice has been developed to complement postgraduate curriculum reflecting how curricula have evolved in relation to health care service and societal needs.[25] Furthermore, the introduction of a UK-wide Medical Licencing Assessment in 2022 may influence what medical schools teach and provide opportunities to keep curricular content relevant and inclusive.

Thus, by examining the evidence for need to change or update medical curricula, and through reflection on content and process, Mezirow's concept of transformative learning could be applied by medical educators in curricular development.[49] For instance in Scotland, the Scottish Deans' Medical Curriculum Group (SDMCG) discusses and helps coordinate the development, delivery and evaluation undergraduate curricula. Their aim is to ensure that graduates from each school in Scotland are of an equivalent standard. Their review of the curriculum has also been informed by frameworks on specialist subjects like acute and emergency medicine, dermatology, neurology, palliative care, pharmacology and sexual health.[50]

The curricula developed for foundation and professionalism related subjects recommended their components be implemented by integration both horizontally and vertically throughout the medical course. These subjects, relevant and applicable to all clinical specialties, could be included in generic outcomes for the specialties and form a framework through the undergraduate years.

It is vital for UG curricula to evolve by keeping abreast with changing clinical needs and advances in diagnostics, investigative and management strategies.

With limited availability of resources and time, the curricula could be split into essential, desirable and nice to know categories to help prioritise areas of teaching and learning. The use of technological advances (e.g. use and sharing of e-learning

For peer review only - http://bmjopen.bmj.com/site/about/guidelines.xhtml

resources/ webinars) could help provide flexibility and autonomy in how these curricula could be implemented and delivered, if followed.

LIMITATIONS OF THE REVIEW

We conducted a comprehensive search for all relevant articles for this study. However, as with limitations seen in scoping reviews, articles or studies pertinent to the review question may have been unintentionally omitted.[3] The level of evidence for the quality of the developed specialty specific core-curricula could not be graded in this search, as done with systematic reviews. However, most curricula were developed with expert consensus and use of a Delphi study, suggesting that efforts were made to establish 'best practice' and core curricular outcomes. The curricular components for individual specialties were variable, making comparisons difficult.

We were nevertheless able to establish common themes and drivers for development of the UG core-curricula for UK medical undergraduates. In addition, the study helped identify gaps in specialty specific curricula like the absence of an overarching RCP curricula, as well as the heterogeneity of the curricular designs between specialties.

CONCLUSIONS

This literature review highlights the status of specialty specific core-curricula for medical graduates in UK. This is the first study to provide a comprehensive overview of the curricula developed by specialties and their recommendations on knowledge and skills expected of all UK medical graduates, in alignment with GMC. The review establishes common themes and drivers for development of core-curricula for UK medical undergraduates. The study identifies gaps in development of specific UG curricula as well as the heterogeneity of the curricular designs between specialties and subjects. It would be interesting to see how medical schools are implementing and assessing these curricula and how that is likely to affect future training.

For peer review only - http://bmjopen.bmj.com/site/about/guidelines.xhtml

Contributorship statement:

All 3 authors have made substantial contributions to the planning, conduct, analyses and interpretation of findings, and reporting of the work described in the article, and have agreed to be accountable for all aspects of the work, its accuracy and integrity. MS is responsible for the overall content as guarantor.

MS, RM and GD with RP formulated the review question.

MS screened the titles and abstracts of retrieved citations and MS and RM assessed the full text. MS, RM and GD resolved any ambiguities about inclusion criteria. RM and MS devised the data extraction proforma. MS extracted data and discussed data items with RM and GD. MS contacted study authors for further information.

RM and MS identified common themes, MS conducted the narrative synthesis with RM and GD.

MS wrote the first draft of the manuscript and revised and amended it with RM and GD who also approved the final version to be published. MS is the corresponding author.

The corresponding author attests that all listed authors meet authorship criteria and that no others meeting the criteria have been omitted.

Funding: None

Conflicts of interest: None

Copyright/ Licence for publication:

The Corresponding Author has the right to grant on behalf of all authors and does grant on behalf of all authors, a worldwide licence to the Publishers and its licensees in perpetuity, in all forms, formats and media (whether known now or created in the future), to i) publish, reproduce, distribute, display and store the Contribution, ii) translate the Contribution into other languages, create adaptations, reprints, include within collections and create summaries, extracts and/or, abstracts of the Contribution, iii) create any other derivative work(s) based on the Contribution, iv) to exploit all subsidiary rights in the Contribution, v) the inclusion of electronic links from

For peer review only - http://bmjopen.bmj.com/site/about/guidelines.xhtml

the Contribution to third party material where-ever it may be located; and, vi) licence any third party to do any or all of the above."

Acknowledgements:

Dr Rakesh Patel, Clinical Associate Professor in Medical Education & Honorary Consultant Nephrologist, Faculty of Medicine & Health Sciences, University of Nottingham for guidance on the review question

Lisa Lawrence, Clinical librarian, Burton and Derby Teaching Hospitals NHS Foundation Trust, Derby for conducting the searches for the review

Prof Cathy Bennett, Royal College of Surgeons in Ireland (RCSI), Office of Research and Innovation. Manuscript revision.

Some of the work has been previously presented as oral presentation at:

British Association of Dermatologist (BAD) Teachers of Undergraduate Dermatology Annual Meeting on 6th March 2018

Association for the Study of Medical Education (ASME) Annual Scientific Meeting on 12th July 2018

Ethical approval: None required

Data availability statement: All data relevant to the study are included in the article or uploaded as supplementary information

For peer review only

For peer review only - http://bmjopen.bmj.com/site/about/guidelines.xhtml

References:

1. Promoting excellence: standards for medical education and training. (2015). Manchester, pp.6, 7. Available at: https://www.gmc-uk.org/-/media/documents/Promoting_excellence_standards_for_medical_education_and_training_0715.pdf_61939165.pdf [Accessed 28 Sep. 2018].

2. Medical Licencing Assessment. (2018). General Medical Council. Available at: https://www.gmc-uk.org/education/standards-guidance-and-curricula/projects/medical-licensing-assessment [Accessed 28 Sep. 2018].

3. Outcomes for graduates. (2018).General Medical Council. Available at: https://www.gmc-uk.org/-/media/documents/dc11326-outcomes-for-graduates-2018_pdf-75040796.pdf [Accessed 28 Sep. 2018].

4. Future Hospital: caring for medical patients. (2013). Available at: https://www.rcplondon.ac.uk/projects/outputs/future-hospital-commission [Accessed 28 Sep. 2018].

5. Arksey H, O'Malley L. Scoping studies: towards a methodological framework. Int J Soc Res Methodol. 2005; 8 (1):19-32.

6. The Royal College of Paediatrics. Undergraduate Curriculum for child health. 2015. http://www.rcpch.ac.uk. [Accessed 3 Apr 2017].

7. Royal College of Obstetrics & Gynaecologists. National Undergraduate Curriculum in Obstetrics and Gynaecology.UK.2015. https://www.rcog.org.uk/en/careers-training/specialty-training-curriculum/undergraduate-training-in-og/ [Accessed 3 Apr 2017].

8. Royal College of Psychiatrists. Core Curriculum in Psychiatry. 2011. http://www.rcpsych.ac.uk/workinpsychiatry/faculties/academic/undergraduateteaching/curriculum.aspx. [Accessed 4 Apr 2017].

9. The Royal College of Surgeons. National Undergraduate Curriculum in Surgery.2015.

For peer review only - http://bmjopen.bmj.com/site/about/guidelines.xhtml

https://www.rcseng.ac.uk//media/files/rcs/library-and-publications/non-journal-publications/undergraduate_curriculum_2015-web.pdf. [Accessed 14 May 2017].

10. Baldock C. The Acute Care Undergraduate TEaching (ACUTE) Initiative: consensus development of core competencies in acute care for undergraduates in the United Kingdom. Intensive Care Med. 2005; 31 (12):1627-33.

11. Linklater GT, Bowden J, Pope L et al. Developing learning outcomes for medical students and foundation doctors in palliative care: a national consensus-seeking initiative in Scotland. Med Teach. 2014; 36 (5): 441-446.

12. Paes P, Wee B. A Delphi study to develop the Association for Palliative Medicine consensus syllabus for undergraduate palliative medicine in Great Britain and Ireland. Palliative medicine. 2008; 22 (4): 360-364.

13. Association of British Neurologists. Undergraduate Neurology.2017. http://www.theabn.org/what-we-do/training/undergraduate-neurology.html [Accessed 14 May 2017].

14. British Association of Dermatologists. Undergraduate Dermatology Curriculum.2016 http://www.bad.org.uk/librarymedia/documents/BAD%202016%20revised%20UG%20Curriculum%20(UK).pdf. [Accessed 14 May 2017].

15. Smith CF, Finn GM, Stewart J, McHanwell S. Anatomical Society core regional anatomy syllabus for undergraduate medicine: The Delphi process. J.Anat. 2016; 228 (1):2-14.

16. The Royal College of Pathologists. Pathology Undergraduate Curriculum. 2014. https://www.rcpath.org/resourceLibrary/pathology-undergraduate-curriculum-.html. [Accessed 3 Apr 2017].

17. Jackson, A.A., Human nutrition in medical practice: the training of doctors. Proc Nut Soc. 2001; 60 (2): 257-63.

For peer review only - http://bmjopen.bmj.com/site/about/guidelines.xhtml

18. Stirrat GM, Johnston C, Gillon R. Boyd K. Medical ethics and law for doctors of tomorrow: the 1998 Consensus Statement updated. J med ethics. 2010; 36 (1):55-60.

19. Masud T, Blundell A, Gordon AL, et al. European undergraduate curriculum in geriatric medicine developed using an international modified Delphi technique. Age and ageing. 2014; 43 (5):695-702.

20. Bachmann C, Abramovitch H, Barbu CG, et al. A European consensus on learning objectives for a core communication curriculum in health care professions. Patient Educ Couns. 2013; 93 (1):18-26.

21. Noble LM, Scott-Smith W, O'Neill B, Salisbury H. Consensus statement on an updated core communication curriculum for UK undergraduate medical education. 2018; 101 (9) 1712-19.

22. British Geriatrics Society Recommended Curriculum in Geriatric Medicine for MedicalUndergraduates.2013. http://www.bgs.org.uk/generalinformation171/secttrainees/generalinfo/undergraduatecurriculum. [Accessed 14 May 2017].

23. Woolf AD, Walsh NE. Global core recommendations for a musculoskeletal undergraduate curriculum. Ann Rheum Dis. 2004; 63:517-24.

24. Coady D, Walker D, Kay L. Regional Examination of the Musculoskeletal System (REMS): a core set of clinical skills for medical students. Rheumatology. 2004; 43: 633–639.

25. Royal College of General Practitioners. (2018).Teaching general practice. Guiding principles for undergraduate general practice curricula in UK medical schools. [Accessed 3 March 2019].

26. The Royal College of Radiologists. Medical undergraduate non-surgical oncologycurriculum.2014. https://www.rcr.ac.uk/clinical-oncology/undergradute-oncology. [Accessed 4 May 2017].

27. The Royal College of Radiologists. Undergraduate radiology curriculum second edition. London, 2017. www.rcr.ac.uk. [Accessed 4 Apr 2017].

For peer review only

For peer review only - http://bmjopen.bmj.com/site/about/guidelines.xhtml

28. Royal College of Ophthalmologists. "Eyes & Vision Curriculum" for Undergraduate and Foundation Doctors. 2015. https://www.rcophth.ac.uk/wp-content/uploads/2014/07/Undergraduate-and-Foundation-doctors-curriculum.pdf.[Accessed 3 Apr 2017].

29. Public Health Educators in Medical Schools. Undergraduate Public Health Curriculum for UK Medical Schools. Consensus Statement 2014. http://www.fph.org.uk/uploads/PHEMS booklet web.pdf. [Accessed 14 May 2017].

30. Ross S, Maxwell S. Prescribing and the core curriculum for tomorrow's doctors: BPS curriculum in clinical pharmacology and prescribing for medical students. Br. J. Clin. Pharmacol. 2012; 74 (4):644-61.

31. Core curriculum for sociology in undergraduate medical education. Behavioural & Social Sciences Teaching in Medicine (BeSST) Sociology SteeringGroup.2016.http://www.ed.ac.uk/usher/news-events/news-2016/core-curriculum-sociology-medical-education. [Accessed 20 Apr 2017].

32. Turner K, Brewster S. Undergraduate urology: A survey of current provisions and guidelines for a core curriculum. BJU Int. 2002; 90 (4): 475.

33. The British Association of Urological Surgeons. An undergraduate syllabus for urology.2013.https://www.baus.org.uk/_userfiles/pages/files/professionals/education/Undergraduate%20Syllabus.pdf. [Accessed 14 May 2017].

34. Lyoyd S, Tan ZE, Taube M-A, Doshi J. Development of an ENT undergraduate curriculum using a Delphi survey. Clin Otolaryngol. 2014; 39:281-288.

35. Bleakley A, Marshall R, Brömer R. Toward an aesthetic medicine: developing a core medical humanities undergraduate curriculum. J med humanit. 2006; 27 (4):197-213.

36. WHO Multi-professional Patient Safety Curriculum Guide.2011. World Health Organization.http://www.who.int/patientsafety/education/mp_curriculum_guide/en/ [Accessed 3 Apr 2017].

For peer review only

For peer review only - http://bmjopen.bmj.com/site/about/guidelines.xhtml

37. Burge SM. Undergraduate medical curricula: are students being trained to meet future service needs? Clin Med.2003; 3 (3): 243-6.

38. Association of British Neurologists Training & Education Subcommittee. Clinical Neurology Medical Undergraduate Curriculum UK Medical School Survey.2002. http://www.theabn.org/media/Med%20Sch%20Neuro%20Survey%202002-3.pdf [Accessed 30/7/14] and CM Wiles, P Bain (Personal communications 2011).

39. Wilkinson IMS. A survey of undergraduate teaching of clinical neurology in the UK 1990. J Neurol Neurosurg Psych. 1991; 54:266-68.

40. General Medical Council (2010) Standards for curricula and assessment systems. 2010. http://www.gmc-uk.org/education/postgraduate/standards_forcurricula_and _assessment_systems.asp. [Accessed 14 May 2017].

41. McIntosh N, Olver RE, Haddad, D. What is core? Guidelines for the core curriculum in paediatrics. Med Educ.1997; 31(5): 354-8.

42. Hirsh D, Gaufberg E, Ogur B, Cohen P, Krupat E, Cox M, Pelletier S, Bor D. Educational outcomes of the Harvard Medical School-Cambridge integrated clerkship: a way forward for medical education. Acad Med. 2012. 87(5): 643-50.

43. Voss JD, May NB, Schorling JB et al. Changing conversations: teaching safety and quality in residency training. Academic Medicine.2008; 83:1080-7.

44. Copeland C, Fisher J, Teodorczuk A. Development of an international undergraduate curriculum for delirium using a modified delphi process. Age and Ageing. 2018; 47. 131-73.

45. Wakefield RJ, Weerasingheb A Tung P, et al. The development of a pragmatic, clinically driven ultrasound curriculum in a UK medical school Medical Teach 2018; 40(6): 600–606.

For peer review only - http://bmjopen.bmj.com/site/about/guidelines.xhtml

46. Ponzer S, Hylin U, Kusuoffsky A et al. Interprofessional training in the context of clinical practice: goals and students' perceptions on clinical education wards. Medical Education. 2004; 38(7):727-36.

47. Anderson ES, Lennox A. The Leicester Model of Interprofessional education: developing, delivering and learning from student voices for 10 years. J Interprof Care.2009; 23(6):557-73.

48. NHS England. General Practice Forward View.2016. https://www.england.nhs.uk/gp/gpfv/ [Accessed 22 May 2017].

49. Mezirow J and associates (eds). Fostering Critical Reflection in Adulthood. 1990. Jossey-Bass, San Francisco, CA.

50. Scottish Deans' Medical Curriculum Group. The Scottish Doctor: Learning Outcomes for the medical undergraduate in Scotland: a Foundation for Competent and Reflective Practitioners. 2008: Dundee. UK. http://www.scottishdoctor.org/resources/scotdoc3.pdf[Accessed 22 May 2017]

For peer review only

TABLE 1: ELECTRONIC DATA COLLECTION TABLE OF SPECIALTY OR SUBJECT WITH CURRICULUM IN ALPHABETICAL ORDER

	Specialty with curriculum	Year	Organization/Body	Alignment with GMC outcomes
**1.**	**Acute care**[10]	2005	Resuscitation Council UK	Y
**2.**	Regional **Anatomy**[15]	2016	Anatomical Society	Y
**3.**	**Clinical pharmacology and prescribing**[30]	2012	British Pharmaceutical Society	Y
**4. a**	**Core communications curriculum**[20]	2013	European study using Delphi process	
**b**	**Communications curricula**[21]	2018	U. K. Council for Clinical Communication Skills Teaching in Undergraduate Medical Education	Y
**5.**	**Dermatology**[14]	2016	British association of dermatologists	Y
**6.**	**ENT**[34]	2014	A Delphi survey (UK) of ENT consultants and specialist registrars, accident and emergency consultants and specialist registrars,	Y

For peer review only - http://bmjopen.bmj.com/site/about/guidelines.xhtml

			general practitioners and paediatricians.	
**7.**	**General Practice**[25]	2018	Royal college of general practitioners	Y
**8. a**	European undergraduate curriculum in **geriatric medicine**[19]	2014	European Union of Medical Specialists	Y
**b**	**Geriatric medicine** for undergraduates[22]	2013	British Geriatrics Society	Y
**9.**	**Medical Ethics and Law** [18]	2010	Medical Education Working Group of the Institute of Medical Ethics and associated signatories	Y
**10.**	**Medical Humanities**[35]	2006	Peninsula medical school	Unclear
**11.a**	**Musculoskeletal system (MSK)**[23]	2004	The Bone and Joint Decade ((BJD) Undergraduate Curriculum Development Group	Y
**b**	**Regional Examination of the Musculoskeletal System (REMS)**[24]	2004	UK representation of the four specialties- Rheumatologists, Orthopaedic	Y

For peer review only - http://bmjopen.bmj.com/site/about/guidelines.xhtml

			Surgeons, Geriatricians and General Practitioners	
**12.**	**Neurology**[13]	2017	Association of British Neurologists	Y
**13.**	Human **Nutrition**[17]	2001	National Nutrition Task Force	Y
**14..**	**Obstetrics & Gynaecology**[7]	2015	Royal College of Obstetricians and Gynaecologists (RCOG)	Y
**15.**	**Ophthalmology**[28]	2015	Royal college of Ophthalmologists	Y
**16.**	**Oncology**[26]	2014	The Royal College of Radiologists	Y
**17.**	**Paediatrics**[6]	2015	Royal college of paediatrics and child health	Y
**18. a**	**Palliative Care**[11]	2014	Scottish Palliative Medicine Curriculum Development Group	Y
**b**	**Palliative Medicine**[12]	2008	Association for Palliative Medicine	Y
**19.**	**Pathology**[16]	2014	The Royal College of Pathologists	Y
**20..**	**Patient Safety**[36]	2011	World Health Organization	Y
**21.**	**Psychiatry**[8]	2011	Royal college of	Y

For peer review only - http://bmjopen.bmj.com/site/about/guidelines.xhtml

			psychiatrists	
**22.**	**Public Health**[29]	2014	Faculty of Public Health	Y
**23.**	**Radiology**[27]	2017	Royal college of radiologists	Y
**24.**	**Sociology**[31]	2016	Behavioural & Social Sciences Teaching in Medicine (BeSST) Sociology Steering Group	Y
**25.**	**Surgery**[9]	2015	Royal college of Surgeons	Y
**26.a**	**Urology**[32]	2002	Journal article in Urology	Unclear
**b**	**Urology syllabus**[33]	2012	British Association of Urological Surgeons	Unclear

For peer review only - http://bmjopen.bmj.com/site/about/guidelines.xhtml

Supplementary material

Figure's legend

Figure 1: PRISMA flow diagram of the scoping review

Appendix 1: Example of search strategy (MEDLINE)

For peer review only

For peer review only - http://bmjopen.bmj.com/site/about/guidelines.xhtml

Figure 1: PRISMA (preferred reporting items for systematic reviews and meta-analysis) flow diagram of the scoping review

UG= undergraduate

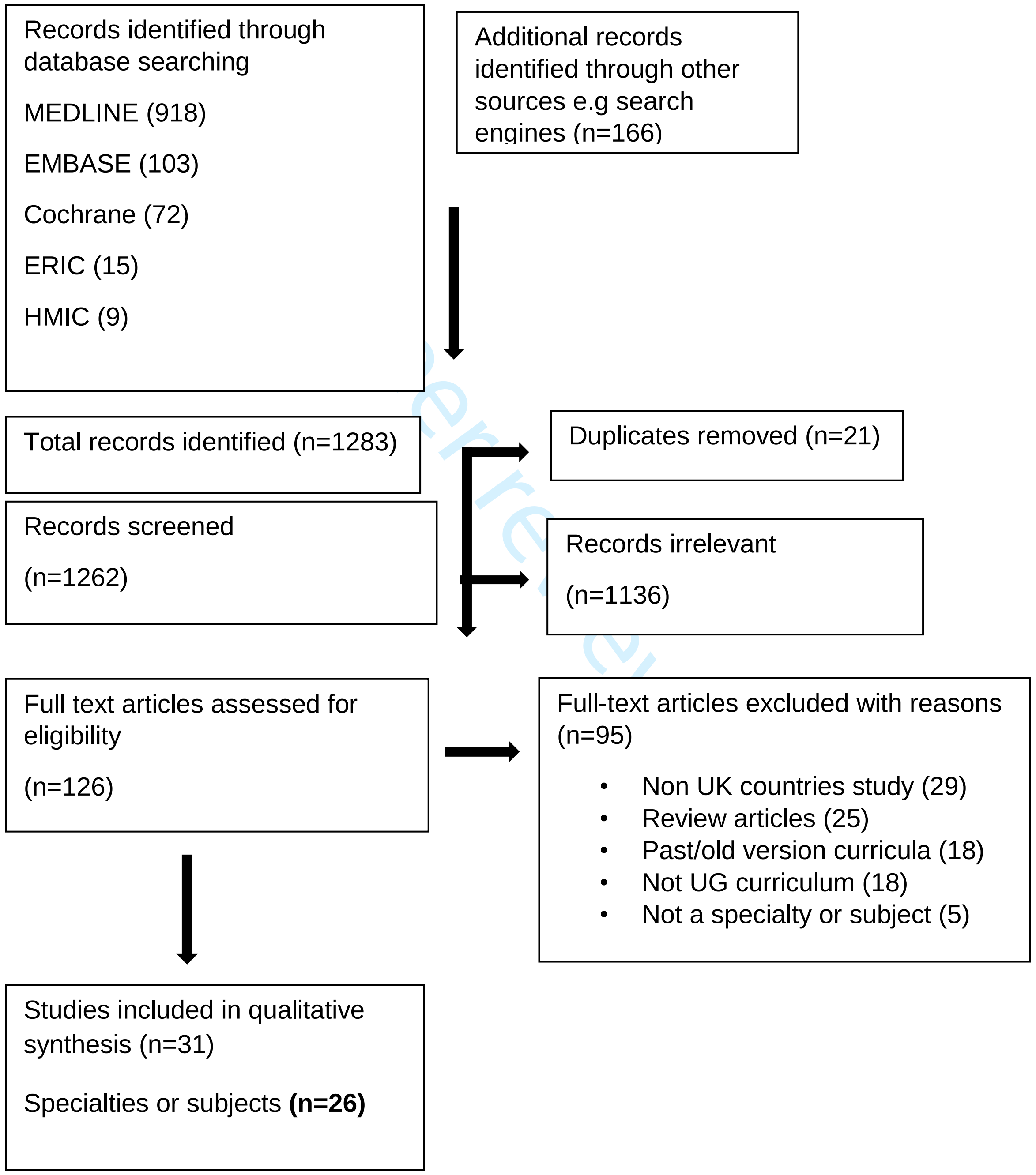

Example Search Strategy for Appendix

NICE Healthcare Databases Advanced Search MEDLINE 1946 to present (Provided by Proquest).

Strategy 606888

#	Database	Search term	Results
1	Medline	"EDUCATION, MEDICAL, UNDERGRADUATE"/	22398
2	Medline	("medical education").ti,ab	36006
3	Medline	(undergraduate).ti,ab	29224
4	Medline	("medical student").ti,ab	6528
5	Medline	"STUDENTS, MEDICAL"/	30311
6	Medline	(2 AND 3)	3401
7	Medline	(1 OR 4 OR 5 OR 6)	48667
8	Medline	CURRICULUM/	70249
9	Medline	(curricul*).ti,ab	48931
10	Medline	(8 OR 9)	93842
11	Medline	(core OR influenc* OR design* OR develop* OR deliver* OR enabl* OR barrier* OR process* OR factor*).ti,ab	9645339
12	Medline	"REFERENCE STANDARDS"/	40365
13	Medline	(standard*).ti,ab	1110534
14	Medline	(12 OR 13)	1133896
15	Medline	(11 OR 14)	10194258
16	Medline	DERMATOLOGY/	17224

For peer review only

17	Medline	(dermatology).ti,ab	23551
18	Medline	(16 OR 17)	33537
19	Medline	exp "UNITED KINGDOM"/	350174
20	Medline	(UK OR "United Kingdom" OR "Great Britain" OR GB OR England OR Wales OR "Northern Ireland" OR Scotland).ti,ab	205051
21	Medline	(19 OR 20)	459845
22	Medline	(7 AND 10 AND 15)	8130
23	Medline	22 [DT 1994-2019]	7323
24	Medline	(7 AND 10 AND 15 AND 18)	57
25	Medline	24 [DT 1994-2019]	52
26	Medline	(7 AND 10 AND 15 AND 21)	788
27	Medline	26 [DT 1994-2019]	718
28	Medline	(7 AND 10 AND 15 AND 18 AND 21)	8
29	Medline	28 [DT 1994-2019]	8

For peer review only

Preferred Reporting Items for Systematic reviews and Meta-Analyses extension for Scoping Reviews (PRISMA-ScR) Checklist

SECTION	ITEM	PRISMA-ScR CHECKLIST ITEM	REPORTED ON PAGE #
**TITLE**			
Title	1	Identify the report as a scoping review.	1
**ABSTRACT**			
Structured summary	2	Provide a structured summary that includes (as applicable): background, objectives, eligibility criteria, sources of evidence, charting methods, results, and conclusions that relate to the review questions and objectives.	2
**INTRODUCTION**			
Rationale	3	Describe the rationale for the review in the context of what is already known. Explain why the review questions/objectives lend themselves to a scoping review approach.	4
Objectives	4	Provide an explicit statement of the questions and objectives being addressed with reference to their key elements (e.g., population or participants, concepts, and context) or other relevant key elements used to conceptualize the review questions and/or objectives.	4
**METHODS**			
Protocol and registration	5	Indicate whether a review protocol exists; state if and where it can be accessed (e.g., a Web address); and if available, provide registration information, including the registration number.	Not applicable
Eligibility criteria	6	Specify characteristics of the sources of evidence used as eligibility criteria (e.g., years considered, language, and publication status), and provide a rationale.	5 and 6
Information sources*	7	Describe all information sources in the search (e.g., databases with dates of coverage and contact with authors to identify additional sources), as well as the date the most recent search was executed.	5 and appendix 1
Search	8	Present the full electronic search strategy for at least 1 database, including any limits used, such that it could be repeated.	5 and appendix 1
Selection of sources of evidence†	9	State the process for selecting sources of evidence (i.e., screening and eligibility) included in the scoping review.	5,6
Data charting process‡	10	Describe the methods of charting data from the included sources of evidence (e.g., calibrated forms or forms that have been tested by the team before their use, and whether data charting was done independently or in duplicate) and any processes for obtaining and confirming data from investigators.	6 and Data from individual studies extracted into Table 1
Data items	11	List and define all variables for which data were sought and any assumptions and simplifications made.	6 and Table 1

For peer review only

SECTION	ITEM	PRISMA-ScR CHECKLIST ITEM	REPORTED ON PAGE #
Critical appraisal of individual sources of evidence§	12	If done, provide a rationale for conducting a critical appraisal of included sources of evidence; describe the methods used and how this information was used in any data synthesis (if appropriate).	Study quality appraisal not applicable.
Synthesis of results	13	Describe the methods of handling and summarizing the data that were charted.	Table 1
**RESULTS**			
Selection of sources of evidence	14	Give numbers of sources of evidence screened, assessed for eligibility, and included in the review, with reasons for exclusions at each stage, ideally using a flow diagram.	5 and PRISMA diagram
Characteristics of sources of evidence	15	For each source of evidence, present characteristics for which data were charted and provide the citations.	Table 1 and page 7
Critical appraisal within sources of evidence	16	If done, present data on critical appraisal of included sources of evidence (see item 12).	Not applicable
Results of individual sources of evidence	17	For each included source of evidence, present the relevant data that were charted that relate to the review questions and objectives.	7-10 (results section)
Synthesis of results	18	Summarize and/or present the charting results as they relate to the review questions and objectives.	7-10 Narrative synthesis of results
**DISCUSSION**			
Summary of evidence	19	Summarize the main results (including an overview of concepts, themes, and types of evidence available), link to the review questions and objectives, and consider the relevance to key groups.	10-13
Limitations	20	Discuss the limitations of the scoping review process.	13
Conclusions	21	Provide a general interpretation of the results with respect to the review questions and objectives, as well as potential implications and/or next steps.	13
**FUNDING**			
Funding	22	Describe sources of funding for the included sources of evidence, as well as sources of funding for the scoping review. Describe the role of the funders of the scoping review.	14

JBI = Joanna Briggs Institute; PRISMA-ScR = Preferred Reporting Items for Systematic reviews and Meta-Analyses extension for Scoping Reviews.

* Where *sources of evidence* (see second footnote) are compiled from, such as bibliographic databases, social media platforms, and Web sites.

† A more inclusive/heterogeneous term used to account for the different types of evidence or data sources (e.g., quantitative and/or qualitative research, expert opinion, and policy documents) that may be eligible in a scoping review as opposed to only studies. This is not to be confused with *information sources* (see first footnote).

‡ The frameworks by Arksey and O'Malley (6) and Levac and colleagues (7) and the JBI guidance (4, 5) refer to the process of data extraction in a scoping review as data charting.

§ The process of systematically examining research evidence to assess its validity, results, and relevance before using it to inform a decision. This term is used for items 12 and 19 instead of "risk of bias" (which is more applicable to systematic reviews of interventions) to include and acknowledge the various sources of evidence that may be used in a scoping review (e.g., quantitative and/or qualitative research, expert opinion, and policy document).

For peer review only - http://bmjopen.bmj.com/site/about/guidelines.xhtml
